# Home cardiotocography for high-risk obstetric care: A prospective observational feasibility study

**DOI:** 10.1371/journal.pdig.0001823

**Published:** 2026-11-06

**Authors:** Jack Le Vance, Victoria Hodgetts Morton, R. Katie Morris, Leo Gurney

**Affiliations:** 1 Department of Applied Health Sciences, School of Health Sciences, College of Medicine and Health, University of Birmingham, Birmingham, United Kingdom; 2 Birmingham Women's and Children's NHS Foundation Trust, Birmingham, United Kingdom; Southeast University, CHINA

## Abstract

Home cardiotocography (CTG) may support remote antenatal surveillance, reduce hospital attendances, and improve patient-centred maternity care. However, United Kingdom (UK) evidence remains limited, and international findings may not be generalisable due to differences in maternity pathways and digital implementation. We therefore evaluated the feasibility, acceptability, usability, and safety of home CTG monitoring in a UK cohort of high-risk pregnant women. In this single-centre prospective feasibility study, 50 English-speaking women with high-risk singleton pregnancies at ≥32 weeks’ gestation were enrolled. Participants received a portable CTG device and were advised to complete one hour of home monitoring weekly, for up to six weeks, or until delivery. Recordings were reviewed synchronously. The primary outcome was completion of 20 minutes of continuous, interpretable monitoring per episode. Secondary outcomes included feasibility, anxiety, acceptability, usability, competency, technological issues, and safety. In total, 204 home monitoring sessions were completed. The primary outcome was achieved in 82.4% (95% Confidence interval (CI), 76.5%-87.0%) of sessions (168/204), with a mean continuous monitoring duration of 34.3 ± 16.8 minutes. Session number was not associated with achievement of the primary outcome (Odds Ratio (OR) 1.18, 95% CI 0.93–1.51; p = 0.19) or monitoring duration (p = 0.16). Maternal demographic characteristics showed no statistically significant association with monitoring competency in the exploratory analysis. Anxiety scores decreased significantly following participation (p = 0.0004), including before and after the first (p = 0.01) and fifth sessions (p = 0.007). Usability was rated highly (mean 77.6; grade B + , 80th percentile). Participants were advised to attend maternity triage following 13 sessions (6.4%). No prespecified adverse outcomes related to device use occurred. Overall, 78% would use home CTG instead of hospital attendance in a future pregnancy. Home CTG monitoring for high-risk pregnancies was feasible, acceptable, usable, with no safety concerns identified in this single-arm cohort. These findings support future implementation, pathway integration and development of effectiveness studies.

## Author summary

In recent years, there have been rapid advancements in digital technology for pregnancy care, enabling monitoring of the fetal heartrate, namely cardiotocography, to be undertaken in the home setting. Evidence for its use within the United Kingdom (UK) is currently limited and literature from other non-UK healthcare settings may not be directly generalisable, due to differences in clinical care pathways and methods of digital implementation. Consequently, we aimed to comprehensively evaluate the use of home CTG in our high-risk pregnant women to understand if it is a feasible, acceptable, usable and without evidence of safety concerns, whilst further understanding if certain patient characteristics influence how well home CTG is performed. After undertaking 204 home monitoring sessions in a cohort of 50 high-risk women, we found that achieving a consistent duration of home CTG monitoring was feasible, whilst further associated with minimal safety and technological concerns. Participants additionally found home CTG highly usable and acceptable. This study supports the integration of home CTG into clinical care pathways and promotes the development of implementation effectiveness trials to better how home CTG can fit into contemporary maternity care.

## Introduction

Cardiotocography (CTG) is a widely utilised antenatal monitoring strategy, enabling healthcare professionals to evaluate fetal wellbeing [1]. It is most commonly employed for pregnancies classifying as high-risk and is typically complimented with several other additional clinical assessments, including blood pressure and temperature measurement. In recent years, the proportion of high-risk obstetric patients has substantially increased. This is largely attributed to the implementation of national initiatives, an ageing maternal population and changes to guideline scopes, which have lowered the diagnostic threshold for high-risk classification [2–6]. In turn, the demand for CTG has risen, which has led to a strain on the outpatient obstetric service. As frequent CTG surveillance necessitates regular attendance to the hospital, this may impose psychological, emotional and monetary burdens on patients [7,8]. Additionally, regular commuting to and from the hospital raises environmental considerations; an important notion following the National Health Service (NHS) endeavour to reach net zero by 2040 [9]. Accordingly, the need to identify and evaluate strategies to mitigate these clinical, logistical and environmental challenges is paramount to enhancing the quality of contemporary obstetric care.

Advancements in digital technology have accelerated the development of remote devices, capable of facilitating clinical assessments beyond the hospital setting [10,11]. A well-established example is home blood pressure monitoring, which is widely established across maternity services and has demonstrated comparative, if not superior clinical outcomes relative to in-office measurements [12]. In recent years, sophisticated devices, such as patient-operated CTG have emerged, capable of conducting accurate home fetal monitoring. Remote digital maternal-fetal monitoring offers several potential advantages, including enhanced patient autonomy and education, reduced hospital attendance and minimising overall economic burden [13,14]. Importantly, home CTG presents opportunities to improve overall access to fetal monitoring services and helps address persistent disparities in obstetric outcomes across pregnant populations [15]. In conjunction, two of the central pillars in the recent 10-year NHS plan include mobilising care into the community and developing digital technologies [16]. Home CTG aligns closely with both strategies, underscoring its relevance as an area for further investigation.

A recent systematic review conducted by our research team highlighted the non-inferiority of home CTG across multiple maternal, perinatal and healthcare usage outcomes when compared to conventional outpatient CTG [17]. Whilst promising, these previous randomised controlled trials, such as the HoTeL trial, have primarily evaluated clinical outcomes and compared home monitoring with inpatient care in healthcare settings not in the United Kingdom (UK) [13]. In contrast, high-risk pregnancies in the UK are commonly managed through outpatient surveillance pathways, limiting the direct applicability of existing evidence to NHS maternity services. As successful implementation is highly dependent on healthcare context, evidence regarding the feasibility, acceptability and practical implementation of home CTG monitoring within the UK still remains significantly limited. Consequently, further evaluation is required to understand how home CTG monitoring may be integrated into routine UK maternity care and to generate the implementation evidence necessary to inform future effectiveness studies and service development. Our initial pilot study demonstrated proof-of-concept in a small cohort [18]; however, further evaluation in a larger and more diverse population was required to better understand technical performance, patient acceptability, maternal anxiety and patient factors associated with successful home monitoring. These implementation outcomes are critical to successful adoption of remote monitoring technologies within routine clinical practice. Therefore, this study aims to provide a more comprehensive assessment of feasibility of home CTG monitoring in women with high-risk pregnancies receiving care within a UK maternity service.

## Methods

### Ethics statement

The study was approved as part of a wider multi work package study by the London - Westminster Research Ethics Committee (REC: 25/LO/0255) on 15^th^ April 2025 and the Health Research Authority (IRAS351514) (ClinicalTrials ID: NCT07124325). Written consent was obtained from all participants prior to any study activity.

### Study design and participants

We conducted a single centre prospective study at the Birmingham Women’s Hospital from 11^th^ June 2025–19^th^ February 2026. The Birmingham Women’s Hospital is the largest maternity unit in the West Midlands, with approximately 8200 deliveries annually, of which 72% are classified as high risk.

Eligibility and recruitment of participants was attained on the Day Assessment Unit (DAU). This is a midwifery run department, with clinician oversight, whereby high-risk pregnancies will attend at scheduled intervals for outpatient monitoring, which includes CTG. Participants were eligible for inclusion if they were aged ≥18 years old, a singleton pregnancy, ≥ 32 weeks gestational age, English-speaking, had a body mass index (BMI) ≤35 kg/m^2^, and classified as a high-risk pregnancy, due to at least one of the following conditions. The conditions comprised: hypertensive disease in pregnancy (gestational hypertension or pre-eclampsia), small for gestational age fetus, fetal growth restriction, recurrent reduced fetal movements, preterm prelabour rupture of membranes, a previous history of stillbirth and obstetric cholestasis. A definition of each included condition can be seen in S1 Table. Participants with multiple pregnancies, fetal abnormalities, ultrasound Doppler abnormalities (defined as raised pulsatility index in the umbilical artery Doppler and/or absent or reversed end diastolic flow in the umbilical artery Dopler and/or raised peak velocity index for vein in the ductus venosus Doppler and/or or low cerebroplacental ratio in the middle cerebral artery Doppler), women with implanted cardiac devices and those with allergies to skin adhesives and/or latex were excluded.

All participants were provided with a participant information leaflet, followed by a comprehensive face-to-face demonstration of the device and its functionality. Participants consenting to study involvement then supplied written informed consent. Following document completion, participants had the opportunity to ask any additional study questions, and a second device demonstration was provided. This gave information on how to locate the fetal heartrate at the start of the session and mechanisms on how to relocate the fetal heartrate if the fetus moved during the monitoring session. The first home monitoring session was scheduled during recruitment, which was at a mutually agreed time between the research team and participant. Participants were aware to continue their routine antenatal care alongside device usage and to only undertake the home monitoring sessions at the times mutually agreed between both parties.

### Procedures

Home computerised CTG monitoring was conducted using the CE marked PregnaBit Pro device (Nestmedic); a battery-operated device weighing 500 grams (g), with a fetal heartrate measurement accuracy of ±5 beats per minute (bpm) versus standard CTG [19]. This portable, lightweight technology was supplied in a travel case, containing a device with a 5-inch touch display, two transducers (measuring the fetal heartbeat and uterine contractility), one bottle of ultrasound gel, two elastic transducer bands, one heart rate clip, one device charger, one charging adapter, a manual, including a step-by-step infographic for device set-up (Fig 1). The transducer measuring the fetal heartrate uses low-intensity pulsed ultrasound technology at a frequency of 1 megahertz (MHz) and a power of < 10 milliwatts per square centimetre (mW/cm^2^). Fetal heart rate is automatically derived from the acoustic signal using periodicity detection and quality control, providing instantaneous FHR values together with a detection reliability indicator. Measurement of uterine contractability involves the conversion of pressure forces exerted on the transducer into a voltage signal, which is measured by the PregnaBit Pro main unit. The signal is converted into units corresponding to a weight of 1.25g each [20]. The transducers were connected to the device via cables, whilst the heart rate clip, attached to the participants finger and connected with the primary device by Bluetooth 4.0 communication. The total weight of the technology was 1200g.

**Fig 1:**
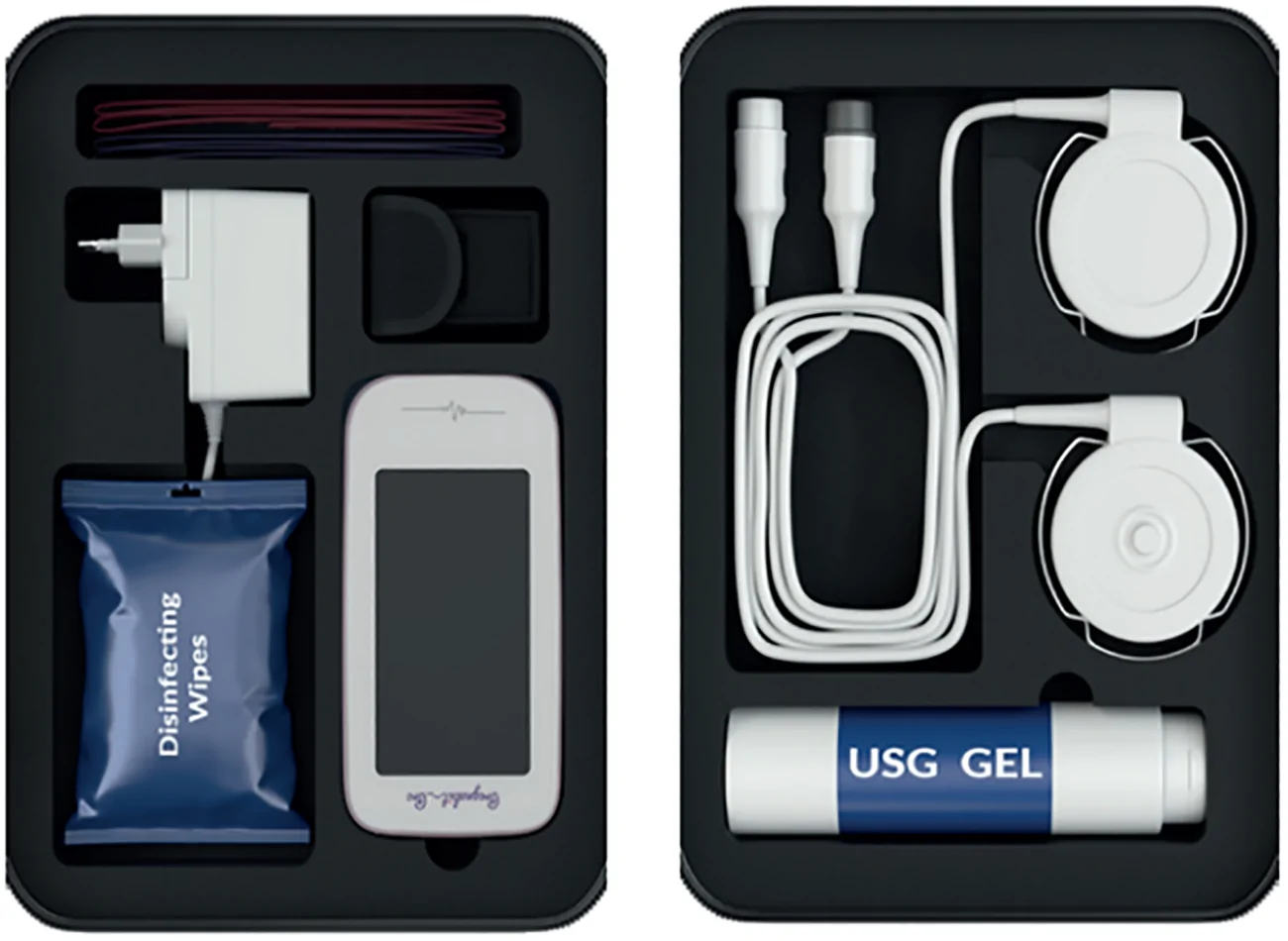
Pregnabit Pro device inside travel case.

Following enrolment, the patient received an email from a dedicated study email address, which contained a selection of summary points for device usage. The email additionally included a link to a 5-minute educational video, developed by the research team, which aimed to promote patient understanding, facilitate device set-up and aid with technical troubleshooting. The patients were encouraged to watch this prior to the first monitoring scheduled. Concurrently, the research team registered the device on the online clinical dashboard.

Registering the device generated a unique pin code which could then be entered on the front screen of the device by the participant to commence the home monitoring session. A different unique pin code was sent one day prior to each monitoring episode via email, in order to minimise unscheduled device usage. The email also facilitated as a participant reminder, by containing the date and time of the monitoring session. Participants were encouraged to charge the device prior to usage, as it required at least 80% battery charge to complete the required monitoring.

Once the unique pin code was entered into the machine, the participants followed a series of instructional videos displayed on the device, including a physical step-by-step infographic to set the device up. Firstly, participants were required to plug both transducer cables into the back of the device and position the transducers both correctly on the abdomen. The participant was advised to secure the transducer measuring uterine contractability on the top of the abdomen with one of the elastic bands provided. Regarding the transducer monitoring the fetal heartrate, the participant was advised to first place the transducer just below the umbilicus and angle it in a circular method to identify the fetal heartrate. If the fetal heartrate was heard in a particular angle, the participant was advised to move the transducer in that direction until the sound of the fetal heartrate, emitting from the device, was at its loudest. If the fetal heartrate was not heard upon angling just below the umbilicus, then the participant was advised to try a different spot on the abdomen, either left of right and repeat the above steps. The device additionally has inbuilt technology to correctly register the presence of a fetal heartbeat, distinguishing from the maternal heartbeat, and indicates correct placement by the changing of a specific icon on the screen from red to green. Once the icon was green, the participant secured the transducer with the other elastic band. Finally, the patient attached the pulse oximeter to their finger. Once complete, the device would commence the monitoring session.

During the monitoring, the participant could visualise a countdown of the monitoring time, the fetal heartrate bpm, the maternal heartrate bpm and the connection strength of both transducers. The participant could additionally press the device screen to record any fetal movements. Concurrently, the monitoring information was synchronously sent via a GSM cellular network to the online clinician dashboard, whereby a computerised CTG was presented for real-time review (Fig 2). Computerised parameters provided included, but not limited to: average baseline fetal heartrate, short term variation (STV) value, long term variation (LTV) value, total high variation time, total low variation time, number of accelerations and number of decelerations. Computerised analysis, including STV was calculated using open source algorithms published by Professor Hans Wolf and followed the same principle as the internationally accepted methodology described by Dawes et al [21–23]. Each monitoring session was reviewed within the hospital by a member of the research team (JLV) with consultant obstetric oversight (VHM, RKM and LG) who were available for all monitoring sessions. Each session was viewed minute-by-minute, however, there was a transmission delay of approximately 15 seconds, which was a pragmatically accepted parameter. In cases where the fetus moved and the transducer signal reduced, the device would instruct the participant to move the device to maintain a clear recording of the fetal heartrate. Despite these measures, if the participant was continuing to have difficulty recording the fetal heartrate, the research team (JLV) could telephone the participant to assist.

**Fig 2:**
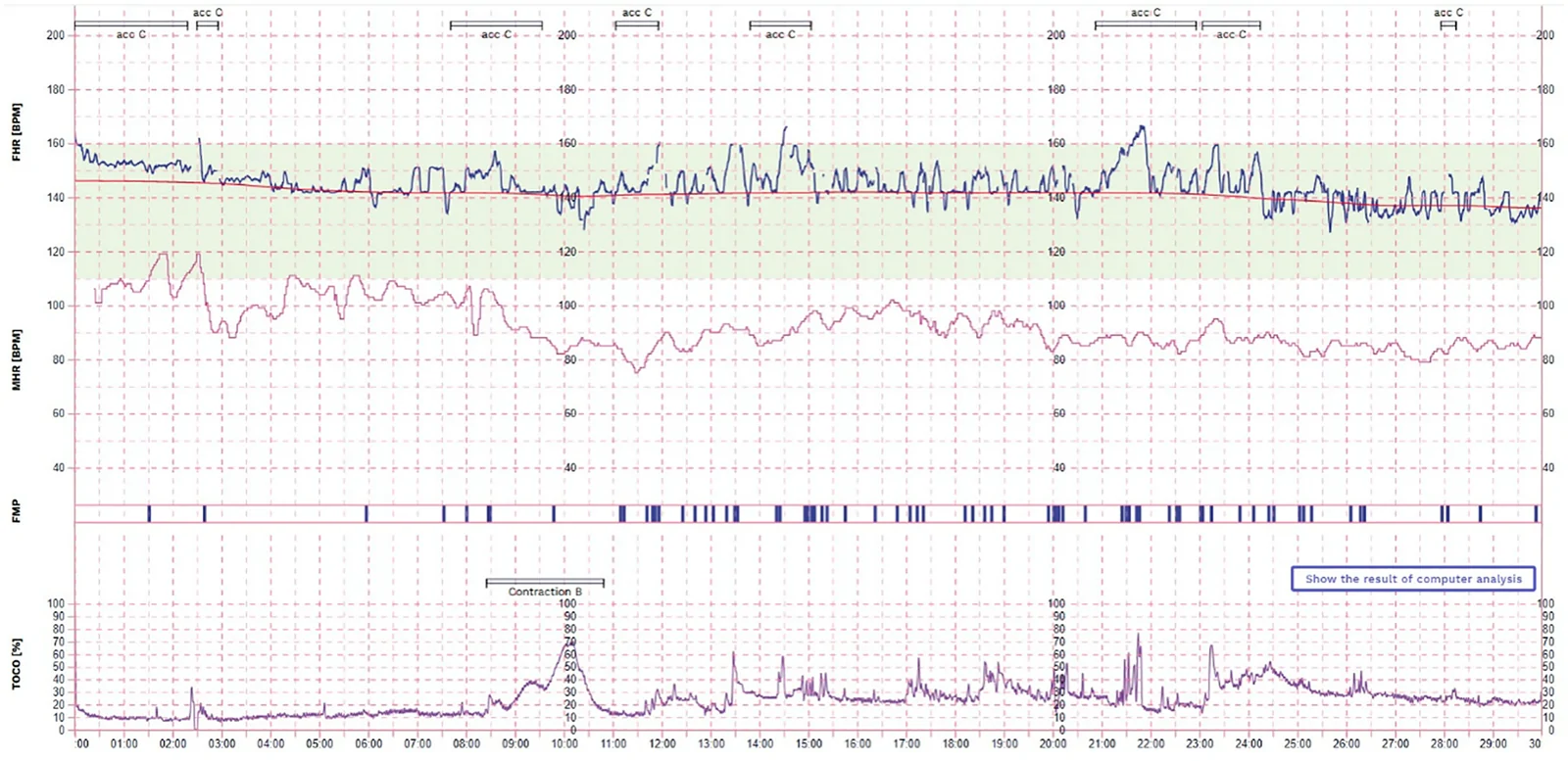
A 30 minute duration of computerized cardiotocography trace visualized on the online clinician dashboard, including the fetal heartrate (blue line), maternal heartrate (pink line), uterine contractability (purple line) and computerized baseline fetal heartrate (red line).

Each participant was advised to complete one hour of home CTG monitoring per week, for up to six weeks, or until delivery. Following completion of each monitoring session, the participant was immediately telephoned by the research team (JLV) to inform of the results. During this time, any questions or device concerns were answered. The next monitoring session was arranged over the phone, and confirmation was sent via email. The participant was able to reschedule a session if needed via email, particularly if it coincided with an unexpected hospital appointment.

Vitally, a strict safety pathway was implemented prior to study commencement. Firstly, if participants had any obstetric concerns, such as lower abdominal pain or reduced fetal movements, they were advised not to commence the remote monitoring, but instead to phone maternity triage. The monitoring session was rescheduled to another time which was suitable for the participant. During each monitoring session, each remote trace was observed for any CTG concerns. Concerns included, a prolonged fetal baseline >160bpm or <110bpm, the presence of decelerations (decrease in baseline fetal heartrate of >15bpm for more than 15 seconds), persistently reduced variability <5bpm or >25bpm, lack of accelerations (increase in baseline fetal heartrate of >15bpm for more than 15 seconds) or abnormal computerised CTG parameters such as a short term variation (STV) <5.0 following the full hour of monitoring. If concerns were present, participants were advised by the research team (JLV) via telephone to immediately present to maternity triage for observations (blood pressure, heart rate, oxygen saturations, respiratory rate and temperature) and a repeat CTG. Participants were reviewed by the research team (JLV) on arrival, who is additionally a member of the obstetric clinical team, and a subsequent plan was made based on the investigation results. Any escalation in care was jointly organised with the on-call clinical team for that day. All enrolled participants additionally had an electronic alert on their maternity notes, which ensured hospital staff were aware of the participants study involvement, in the event of an emergency hospital attendance.

Following study participation completion, the equipment was collected by the research team either in the antenatal or postnatal period, depending on whether the participant had delivered prior to completing all remote monitoring sessions.

### Outcomes

The primary outcome was completion of 20 minutes of continuous, interpretable CTG for each monitoring episode, defined as monitoring competency. The duration was chosen as it is clinically meaningful in traditional clinical care, with patients frequently requiring this duration to enable assessment of the overall trace. The primary outcome was measured overall and additionally across the individual session as an event number, whilst further measuring the exact duration of each home monitoring. Small periods of signal drop lasting <10 seconds whereby the interpretability of the monitoring was not interrupted was pragmatically accepted by the research team. Secondary outcomes evaluated recruitment rate, participant retention, adherence, technical adequacy, anxiety management, acceptability, usability, technological issues and safety. Recruitment rate was defined by the number of eligible participants approach to recruit the designated sample size. Participant retention was measured by the number of participants which exited the study early both due to self-exiting and those withdrawn by the research team. Adherence was determined by the difference between the time scheduled and the time begun for each monitoring session. Technical adequacy was exploratorily evaluated by understanding the influence of demographic and socioeconomic factors on clinically adequate data acquisition. The influences of fetal movements on technical adequacy was also assessed. Anxiety was evaluating using the State Trait Anxiety Inventory (STAI) questionnaire; a widely validated 20-item questionnaire for assessing anxiety in pregnancy [24]. The total score ranges from 20 to 80, with higher scores indicating more significant anxiety. Anxiety was assessed pre- and post-study, whilst additionally assessing a discriminative change in anxiety between individual monitoring episodes (first, third and fifth session). Acceptability was evaluated by pre-study and post-study questionnaires with questions informed by the framework of acceptability by Sekhon et al [25]. Questions were asked on a 5-point Linkert scale (strongly agree to strongly disagree), with additional free text questions. Usability was quantitatively measured using the validated System Usability Scale (SUS); a 10-item questionnaire, with scores ranging from 0-100 [26]. Higher scores indicate greater device usability, and this questionnaire was provided to each participant at the end of device usage. Technological issues, such as device related errors, transmission delays and failures were jointly ascertained through real-time synchronous review of each monitoring session and by participant feedback following each monitoring session. Safety was assessed descriptively via two pre-specified mechanisms. Firstly, by recording the frequency and reasons for any unscheduled maternity triage attendances following each synchronous home CTG monitoring session. And secondly, by documenting any associated maternal or neonatal adverse events relating to device usage, such as emergency caesarean section for acute fetal compromise, admission to the neonatal intensive care unit (NICU), perinatal death or any device-related physical harm (e.g., skin irritation at the transducer site). Any other additional concerns could be directly informed to the research team during each post-monitoring telephone call. As a single-arm feasibility study, any descriptively collected safety data was used to identify potential home CTG safety concerns and inform the design and sample size of future studies. The assessment of safety relative to standard in-clinic monitoring was not within the scope of this study.

### Statistical analysis

Raw data were reported as frequencies, either as percentages, mean and standard deviation (SD) or median and interquartile range (IQR). Achievement of the primary outcome across all sessions was evaluated using logistic mixed-effects model, with participant-specific random intercepts and monitoring session number as a fixed effect. As home CTGs were not independent and participants completed several individual monitoring sessions, a mixed effects method was used to account for within-participant clustering and variation in the number of sessions completed per participant. Results were presented as odds ratios (OR) with 95% confidence intervals (CI). The association between session number and duration of continuous monitoring, or session number and monitoring adherence were analysed using a linear mixed-effects model with random intercepts. Differences in anxiety scores was compared using the Wilcoxon matched-pair test. Results were graphically represented using GraphPad PRISM 7.05 (GraphPad Software, La Jolla, CA, USA). A P value <0.05 was considered statistically significant. Pre- and post-study questionnaire responses were compared using the Chi-squared test to compare the change in proportion of participants selecting strongly agree/agree responses. The Fisher exact test was employed when an event count was five or less.

In order to identify the influence of maternal and sociodemographic factors on the technical adequacy of home data acquisition, determined by achievement of the primary outcome, a multivariable mixed effects logistic regression was employed. The number of fixed-effect parameters was also restricted a priori to avoid model overfitting in this feasibility sample. Models includes a random intercept for participant and were adjusted for maternal age, parity, BMI, ethnicity and education level. Education level was determined by UK scale of education (level 1 (functional skills) to level 8 (doctorate), excluding entry level qualifications) [27]. Due to small numbers within individual ethnic groups and education levels, ethnicity was dichotomised as White British versus non–White British and education dichotomised to level ≥6 (university degree or higher) and <6 (lower than university degree). Parity was additionally dichotomised to nulliparous and multiparous. Maternal age and BMI were modelled as continuous variables to maximise statistical power. The final model was deliberately parsimonious to minimise model completely and avoid overfitting, given the number of participants contributing repeated CTG recordings. Statistical analyses was performed using R version 5.1. The model was fitted using the glmer() function from the lme4 package with a binomial distribution and logit link function; a widely used and extensively validated model for fitting generalised linear mixed-effects models within medical research [28]. Wald tests were used to assess statistical significance of fixed effects. Model estimates were presented with an OR with 95% CI.

Two sensitivity analyses were performed to evaluate the robustness of the primary regression findings. Firstly, a standard logistic regression model was fitted without adjustment for within-participant clustering, treating each CTG session as independent. This ignored the repeated measures structure. Secondly, the primary mixed-effects model was refitted using the BOBYQA optimisation algorithm (Bound Optimisation BY Quadratic Approximation) via the glmerControl() function in R. BOBYQA is a derivative-free optimisation method that is frequently recommended for mixed-effects models because it can improve convergence stability and parameter estimation, particularly in datasets with relatively small to moderate sample sizes or unbalanced clustering [29]. Consistency of effect estimates across models were interpreted as evidence of numerical stability and robustness of findings.

To detect a 20% difference in mean anxiety scores pre- and post-study, with a power of 90% (α = 0.05), a sample size of 42 participants was required. Fifty participants were recruited to account for an approximate 10–15% drop out rate before monitoring sessions were commenced.

### Patient and public involvement

This study was informed by a Patient and Public Involvement and Engagement group (PPIE). The group consisted of an ethnically diverse cohort who were involved in our prior study on home monitoring. The group advised on recommended changes to the protocol, co-designed participant-facing materials and co-developed the educational video for device set-up.

## Results

Between 11^th^ June 2025 and 19^th^ February 2026, a total of 62 high-risk women were approached to recruit 50 participants to this study, representing a recruitment rate of 80.6%. Six participants exited following enrolment, but prior to undertaking the first home monitoring session: attributed by four participants self-exiting (insufficient time commitment (n = 2), anxiety provoking (n = 1) and external family reasons (n = 1)) and two participants exited by the research team (subsequent inpatient admission until delivery (n = 1) and abnormal fetal Doppler (n = 1)). The remaining 44 participants completed a total of 204 home CTG sessions (Fig 3). The mean age of all participants was 31.8 years, and their mean BMI was 27.1 kg/m^2^. The most common high-risk conditions for participant enrolment included recurrent reduced fetal movements (n = 12, 24%) and fetal growth restriction (n = 9,18%). Participants of non-white British ethnicity formulated half of the cohort and 70% had at least a university degree level education. Nearly half of all participants resided in the lowest three deprivation deciles, delineated by home postcode. A full description of the baseline characteristics can be seen in Table 1.

**Table 1:** Baseline characteristics.

Demographic		Home CTG group
Age (mean(SD))		31.8 (5.3)
Gravida (median (IQR))		3 (2-4)
Parity* (median (IQR))		1 (0-2)
BMI (mean(SD))		27.1 (3.6)
Ethnicity	White British	25 (50%)
	Asian	15 (30%)
	Black African	7 (14%)
	White European	3 (6%)
Gestational age at start of monitoring (weeks) (median (IQR))		33 + 0 (32 + 2 – 33 + 4)
Education level	1 – Lowest	3 (6%)
	2	4 (8%)
	3	6 (12%)
	4	1 (2%)
	5	1 (2%)
	6	23 (46%)
	7	11 (22%)
	8 – Highest	1 (2%)
Distance from home to hospital (miles) (mean (SD))		5.8 (2.6)
Duration of enrolment (days) (median (IQR))		26 (10-33)
Deprivation decile^	1 – Least deprived	2 (4%)
	2	2 (4%)
	3	4 (8%)
	4	5 (10%)
	5	6 (12%)
	6	4 (8%)
	7	3 (6%)
	8	3 (6%)
	9	6 (12%)
	10 – Most deprived	15 (30%)
Diagnosis and reason for inclusion in study	Recurrent reduced fetal movements	12 (24%)
	Fetal growth restriction	9 (18%)
	Obstetric cholestasis	9 (18%)
	Hypertensive disease (GHTN or PET)	7 (14%)
	Previous stillbirth	6 (12%)
	Small for gestational age	5 (10%)
	Preterm prelabour rupture of membranes	2 (4%)

*Excluding termination of pregnancy and miscarriage (e.g., G3P1+1). ^Deprivation was delineated by postcode and converted into deciles using the Gov.uk website [30].

GHTN, IQR, interquartile range; gestational hypertension; PET, pre-eclampsia; SD, standard deviation.

**Fig 3:**
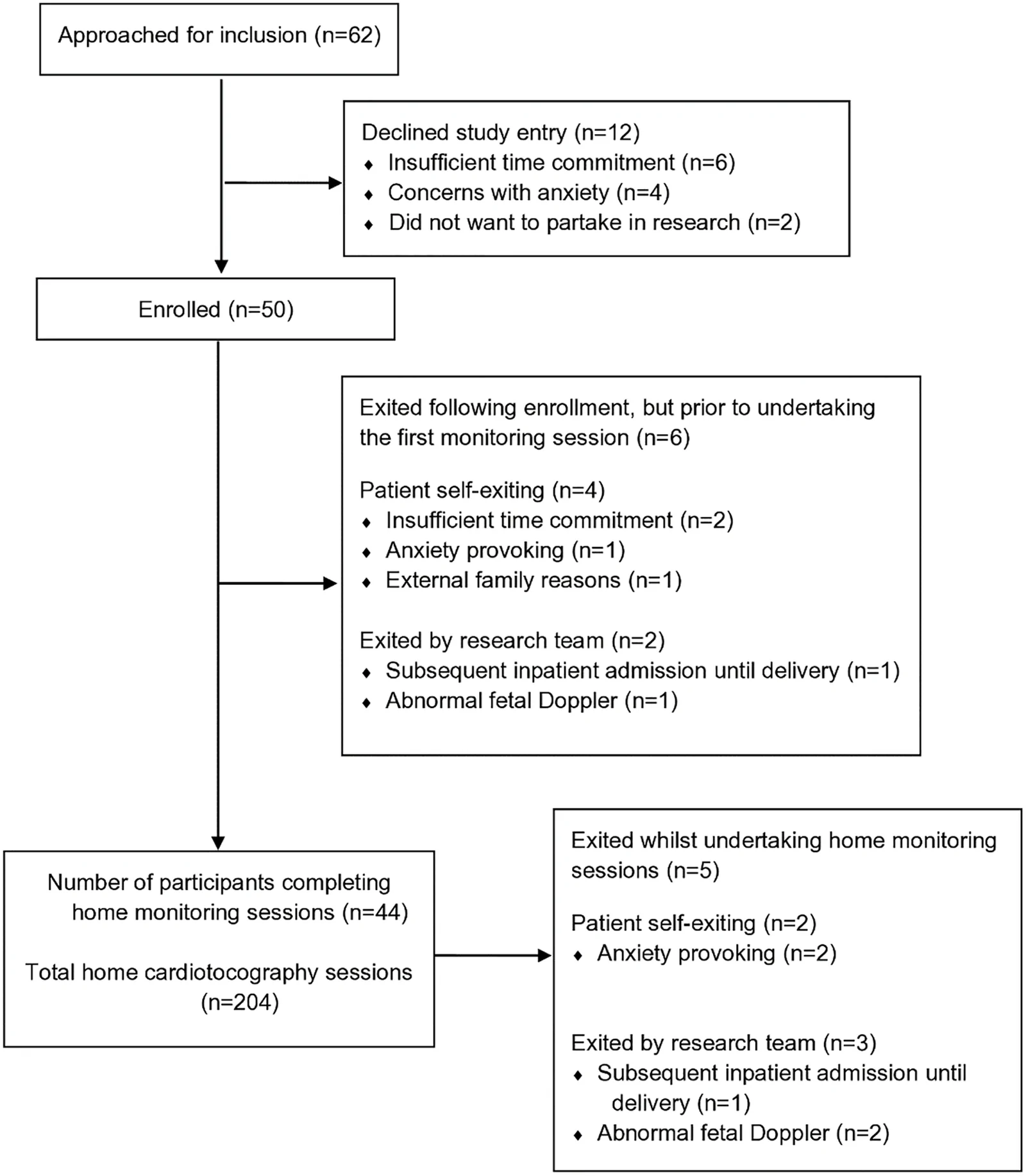
Recruitment flowchart.

Across all participants, including those exiting early, the median number (IQR) of individual remote CTG monitoring’s completed was 5 (2–6), with 38% of participants completing all six sessions. The primary outcome of 20 minutes of continuous, interpretable home CTG monitoring was achieved in 82.4% (95% CI, 76.5%-87.0%) of all sessions (n = 168/204). The mean length of continuous monitoring across all sessions was 34.3 ± 16.8 minutes, with 14.2% of home episodes providing continuous monitoring for the entire 60 minutes. Between the first to the sixth monitoring sessions, episodes sustaining at least 20 minutes of continuous home CTG increased from 81.8% (n = 36/44) to 94.7% (n = 18/19), with an increase in the mean duration of continuous monitoring from 32.3 ± 15.7 minutes to 39.0 ± 15.1 minutes. However, when accounting participants completing differing numbers of home CTG monitoring’s, due to dropout or early delivery, the number of sessions was not a significant predictor for achievement of the primary outcome (OR 1.18, 95% CI 0.93,1.51, p = 0.19), or for increased duration of continuous home CTG monitoring (p = 0.16) (Fig 4) (Table 2). The study dataset can be found in the S2 Table.

**Table 2:** Feasibility outcomes.

Outcome		Home CTG group
**Primary outcome**: 20 minutes of continuous, interpretable monitoring % (95% CI)		82.4 (76.5-87.0))
Home monitoring sessions completed (mean(IQR)		5 (2-6)
Number of home monitoring sessions completed per participant (n(%))	0	6 (12)
	1	3 (6)
	2	4 (8)
	3	2 (4)
	4	7 (14)
	5	9 (18)
	6	19 (38)
Number of sessions achieving the primary outcome stratified by monitoring session (n(%))	1^st^	36/44 (81.8)
	2^nd^	29/41 (70.7)
	3^rd^	32/37 (86.5)
	4^th^	29/35 (82.9)
	5^th^	24/28 (85.7)
	6^th^	18/19 (94.7)
Length of continuous monitoring across all sessions (mean(SD))		34.3 ± 16.8
Number of monitoring sessions stratified by length of continuous monitoring (n(%))	0-9 minutes	15 (7.4)
	10-19 minutes	21 (10.3)
	20-29 minutes	56 (27.5)
	30-39 minutes	37 (18.1)
	40-49 minutes	27 (13.2)
	50-59 minutes	20 (9.8)
	60 minutes	29 (14.2)
Length of continuous monitoring stratified by monitoring session (mean(SD))	1^st^	32.3 ± 15.7
	2^nd^	32.5 ± 18.9
	3^rd^	35.2 ± 15.8
	4^th^	34.9 ± 17.1
	5^th^	35.0 ± 17.0
	6^th^	39.0 ± 15.1

CI, confidence interval; CTG, cardiotocography; n, number; SD, standard deviation.

**Fig 4:**
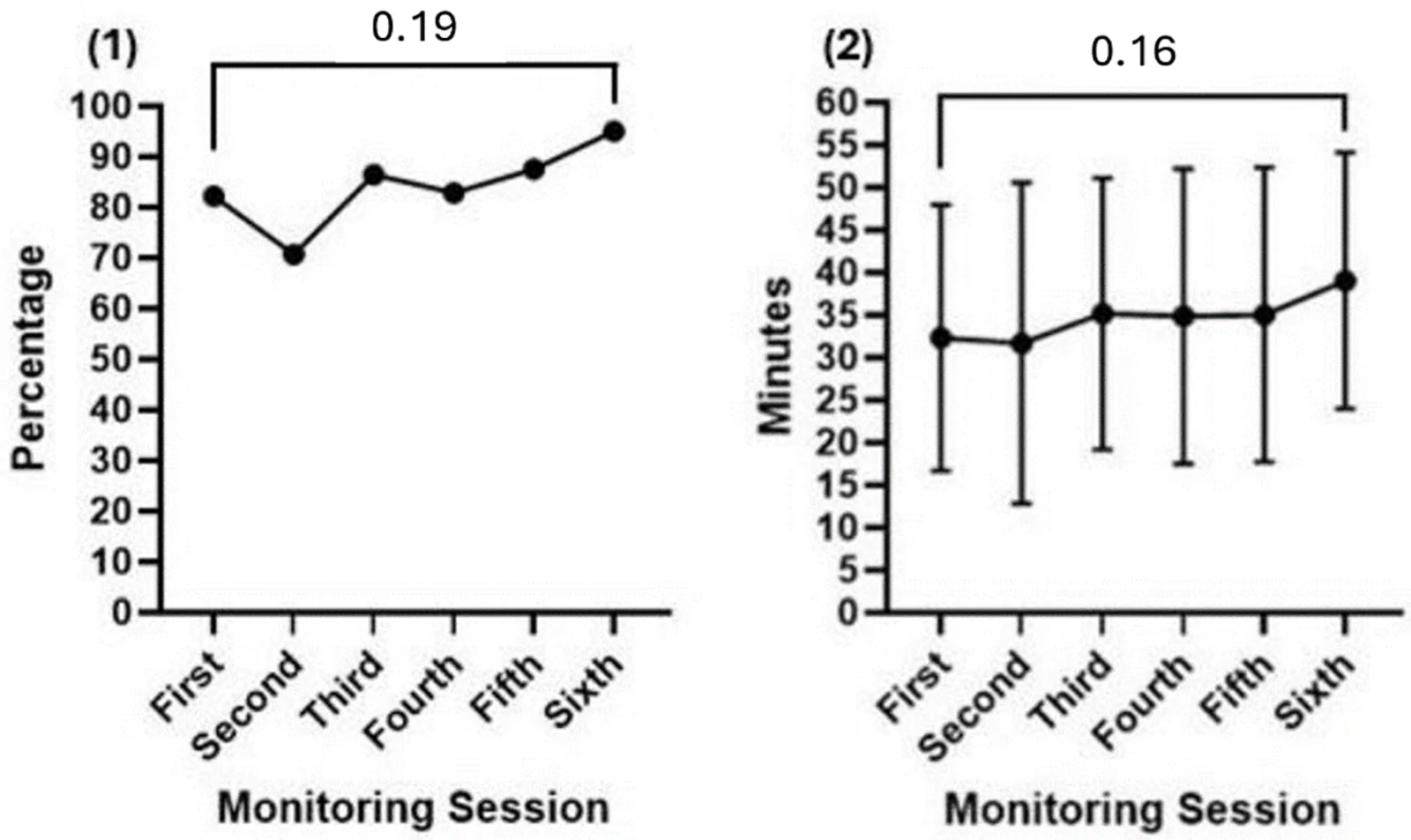
(1) Achievement of 20 minutes of continuous home monitoring as a percentage, delineated across each session. Statistical analysis performed using logistic mixed-effects analysis, with corresponding p value. (2) Duration continuous home monitoring in minutes, stratified by session number. Each session presented with mean and standard deviation. Statistical analysis performed using linear mixed-effects analysis, with corresponding p value.

Participant adherence to home monitoring was consistent and high, with monitoring sessions commencing within 8.2 ± 15.7 minutes from the pre-arranged start time. Monitoring session number was not a significant predictor for maintaining adherence (p = 0.48). In 23 cases (11.3%), there was an insufficient length of continuous home CTG monitoring, primarily due to extensive loss of contact, to enable computerisation and subsequently generate a STV value.

Anxiety was assessed using the STAI questionnaire at several time points including pre and post-study, whilst prior to and directly following the first, third and fifth monitoring sessions. Pre and post-study device usage, there was a statistically significant reduction in anxiety scores (37.5 ± 11.7 versus 31.6 ± 12.0, p = 0.0004) (Fig 5). Anxiety was further significantly reduced when measured prior and directly following the first (35.7 ± 11.0 versus 30.4 ± 11.4, p = 0.01) and fifth monitoring sessions (32.8 ± 11.8 versus 27.3 ± 7.7, p = 0.007), albeit not the third monitoring session (32.3 ± 11.6 versus 29.0 ± 9.4, p = 0.11) (Table 3). When stratifying changes in anxiety across all measured monitoring episodes (1^st^, 3^rd^ and 5^th^ sessions) with respect to achievement of the primary outcome, for episodes whereby <20 minutes of continuous CTG monitoring was attained, there was a non-significant change in anxiety (37.8 ± 11.2 versus 32.6 ± 10.3, p = 0.29), whilst episodes achieving ≥20 minutes was statistically significant (33.2 ± 11.2 versus 28.6 ± 9.6, p = 0.0001). Device usability was further evaluated using the SUS questionnaire. The mean SUS was 77.6 ± 14.3, indicating very good usability (grade B + , 80^th^ percentile).

**Table 3:** Anxiety scores.

Monitoring session		Anxiety score	P value
Pre-study		37.5 ± 11.7	**0.0004**
Post-study		31.6 ± 12.0	
Pre-first monitoring session		35.7 ± 11.0	**0.01**
Post-first monitoring session		30.4 ± 11.4	
Pre-third monitoring session		32.8 ± 11.8	0.11
Post-third monitoring session		27.3 ± 7.7	
Pre-fifth monitoring session		32.3 ± 11.6	**0.007**
Post-fifth monitoring session		29.0 ± 9.4	
Session not achieving the primary outcome	Before	37.8 ± 11.2	0.29
	After	32.6 ± 10.3	
Session achieving the primary outcome	Before	33.2 ± 11.2	**0.0001**
	After	28.6 ± 9.6	

A multivariable logistic mixed-effects model was employed to examine predictors of technical adequacy in home CTG monitoring, with respect to the primary outcome. The analysis included all 204 monitoring sessions from 44 participants. The random intercept variance (SD 0.76) indicated moderate between-participant variability in the baseline probability of successful monitoring.

**Fig 5:**
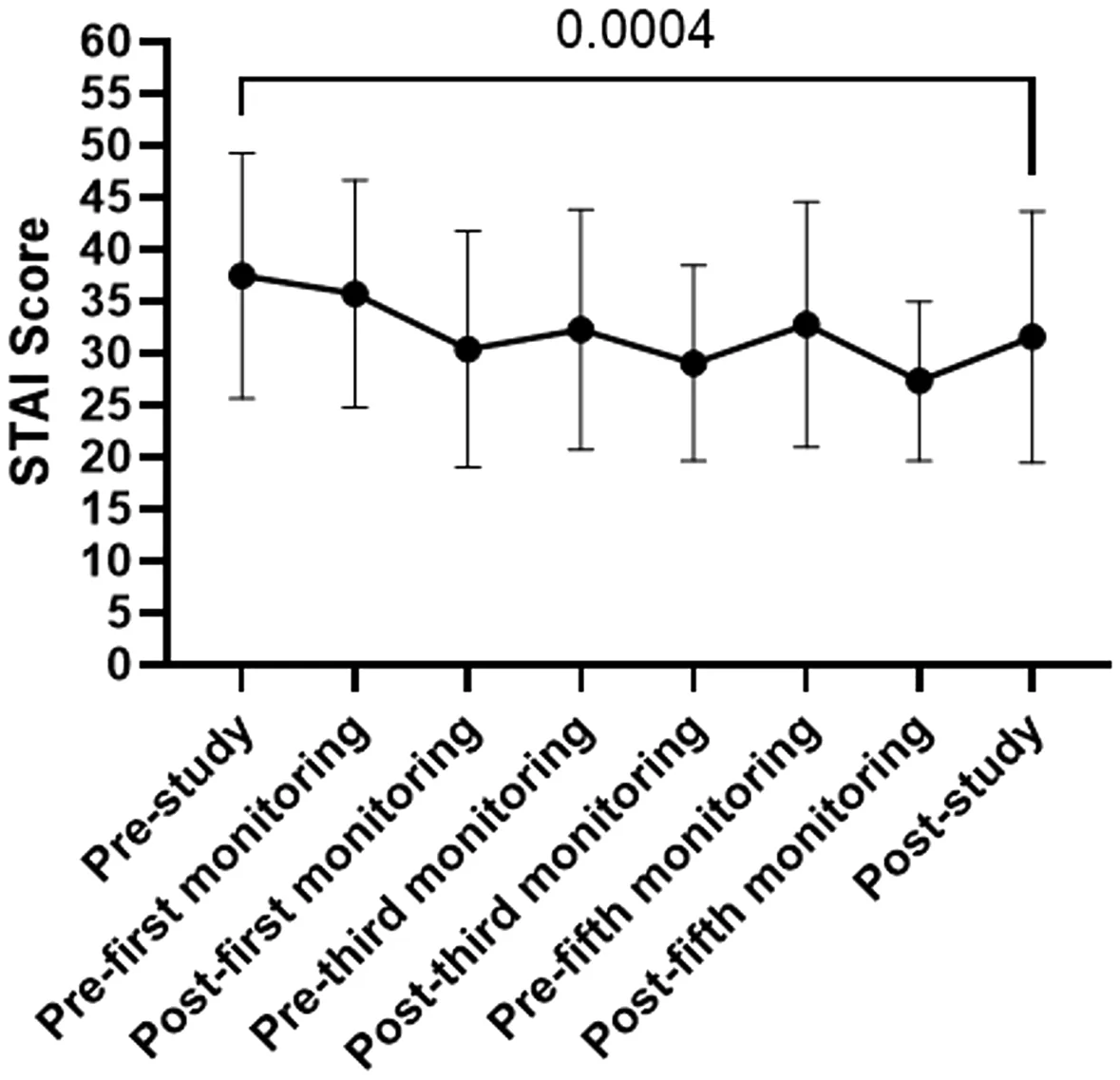
Mean and standard deviation of anxiety scores, stratified by time point, Statistical analysis performed by Wilcoxon signed rank test with corresponding P value for pre-study and post-study score comparison.

Following adjustment for all included parameters, there was no statistically significant association between maternal characteristics and competency of home CTG monitoring. Maternal age showed a weak negative association with successful monitoring (OR 0.91, 95% CI 0.82-1.01; p = 0.09). A full breakdown of results can be seen in Table 4. Additionally, Isolated binary mixed-effects analysis of fetal movements on technical competency further demonstrated no significance (OR 1.00, 95% CI 0.99-1.02; p = 0.56).

**Table 4:** Multivariable logistic regression analysis of socioeconomic variables on achievement of the primary outcome.

Factor	OR	95% Confidence interval		P value
		Lower	Upper	
Age	0.91	0.82	1.01	0.09
BMI	1.00	0.88	1.14	1.00
Ethnicity	1.14	0.41	3.16	0.80
Parity	1.45	0.53	3.97	0.47
Education	1.49	0.46	4.85	0.51

OR, odds ratio.

Employing a standard logistic regression model which did not account for within-participant clustering produced estimates that were highly consistent with the primary mixed-effects model. Effect sizes wee similar in both magnitude and direction for all parameters examined. Results obtained using BOBYQA were virtually identical to those form the primary model. Estimated odds ratios, confidence intervals, and p-values showed negligible differences across all covariates. A visual forest plot comparing all three modelling approaches can be seen in Fig 6. Numerical OR and 95% CI comparison, with associated P values can be seen in the supplementary data (S3 Table).

**Fig 6:**
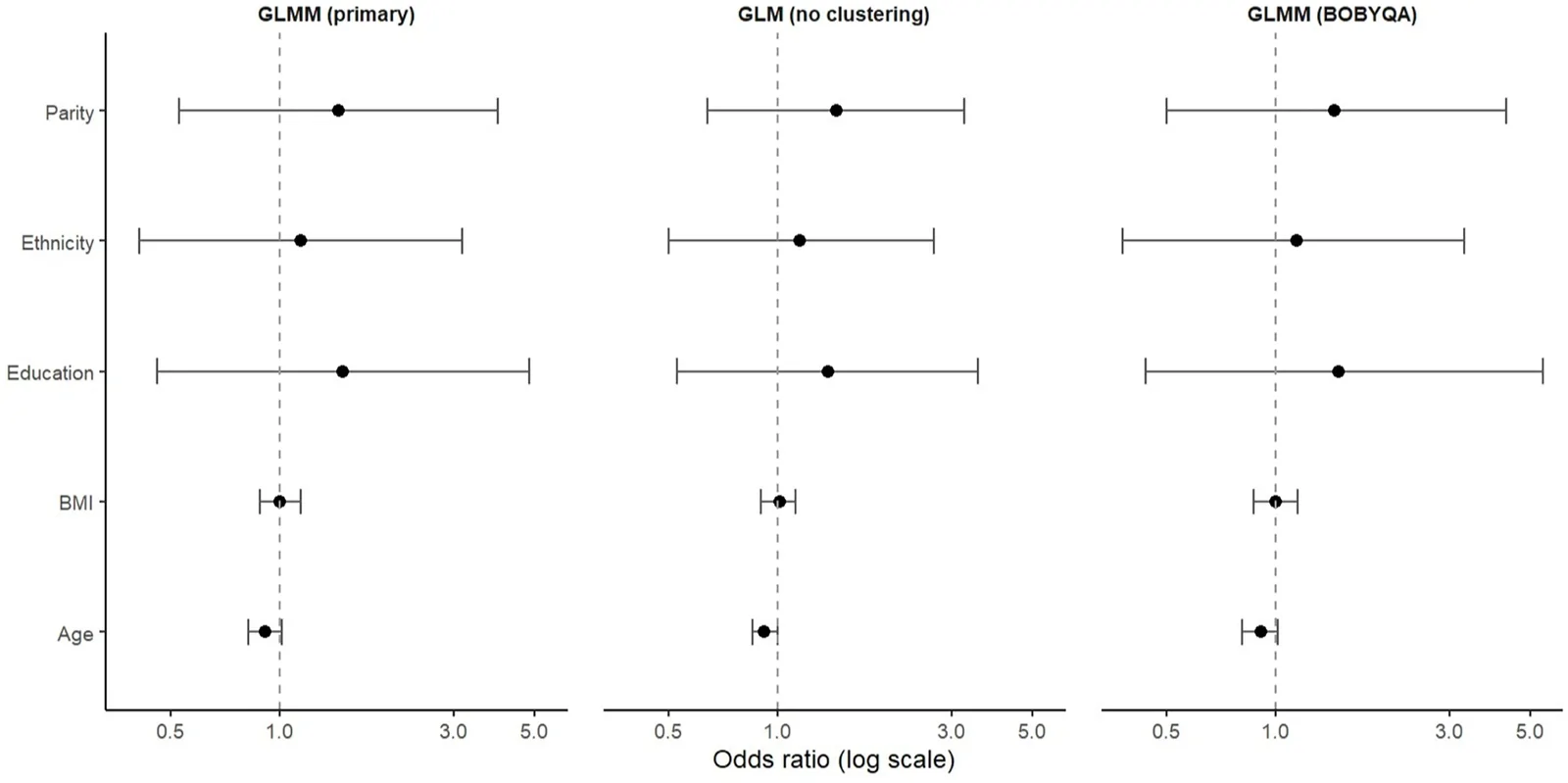
Forest plot showing odds ratios (ORs) and 95% confidence intervals (CIs) across three modelling approaches: (left) the primary generalised linear mixed model (GLMM) with mixed-effects logistic regression, generalised linear model (GLM) logistic regression without clustering, and a mixed-effects logistic regression model fitted using the Bound Optimization BY Quadratic Approximation (BOBYQA) optimisation algorithm. Estimates are highly consistent across models, indicating robustness to both clustering assumptions and the choice of optimisation method.

In 13 (6.4%) home monitoring sessions, 10 participants were asked to attend maternity triage for further assessment. The primary reason for triage attendance included an STV value <5ms (n = 7, 3.4%). In these seven cases, four cases (2.0%) additionally had no episodes of high variation, one case (0.5%) concurrently had reduced variability at the end of the monitoring, one case (0.5%) additionally had a raised fetal baseline heartrate at the end of the monitoring, and one case (0.5%) was otherwise normal with an isolated STV value <5ms. The remaining six cases (2.9%) referred to maternity triage comprised of: the presence of a single or multiple decelerations in the last 20 minutes of monitoring with a normal STV value (n = 3, 1.5%), a raised fetal baseline heartrate at the end of the monitoring with a normal STV value (n = 2, 1.0%) and a low fetal baseline heartrate at the end of the monitoring with a normal STV value (n = 1, 0.5%). In 10/13 cases, a repeat CTG and full set of observations were normal, and the participants were sent home. In one case, a repeat CTG continued to demonstrate an STV value <5ms. The participant attended the following day for another CTG which was normal and was subsequently discharged home. In the final two cases, each participant had repeat CTGs which were normal, however, were admitted to hospital due to an uncontrolled high blood pressure and vaginal bleeding respectively. No pre-specified maternal or perinatal adverse outcomes were recorded in relation to device use during the monitoring period.

Excluding attendances prompted by home CTG monitoring, there were 37 maternity triage attendances among 44 participants during the home monitoring period. The median (IQR) number of triage attendances per participant was 1 (0–1), with 21 participants having no triage attendances. The most common indication for attendance was reduced fetal movements (14/37), followed by abdominal pain (8/37), vaginal bleeding (7/37), headache (4/37), suspected PPROM (2/37) and pruritus (1/37).

Five home sessions (2.5%) initially did not complete the full hour of monitoring due to device-related issues. In three sessions the device self-terminated the monitoring early without a system-reported cause (no error code or fault log was retrievable); one session had insufficient battery charge (user-related as the pre-session charging instructions were not followed); and one session had no sound emitting from the device (hardware-related and on subsequent device inspection, no persistent fault was identified). All monitoring sessions were repeated later that day without any technological issues and replaced the index monitoring session in subsequent analysis. There were no transmission failures across all monitoring sessions, however, a single monitoring session was noted to have a transmission delay of approximately 20 minutes during the final half of the monitoring session. This was attributed to a transient cellular network connectively issue (environmental) rather than a device fault.

Two sessions (1.0%) were accidently terminated by participants, both within five minutes of starting the session, which were immediately restarted without issue. A further single monitoring session (0.5%), included in the analysis, was terminated at 12 minutes by the participant due to back pain. No equipment was lost or damaged during the study period.

Questionnaire responses are detailed within S4 Table. Overall, 84% of participants found the home CTG device acceptable/completely acceptable and 78% agreed that they would be happy to only use this device rather than coming into hospital for the same monitoring during their next pregnancy. Following device completion, the frequency of participants stating the home CTG device required minimal effort to use significantly increased from 44% to 72% (p = 0.002). Themes regarding the benefits and concerns of home fetal monitoring, provided by longer text answers, can be seen in Table 5. Participants frequently highlighted more than one benefit of home monitoring. Commonly cited benefits include reassurance, a reduction in travel, facilitating convenience and saving cost. Concerns raised included difficultly locating the fetal heartbeat, particularly when the fetus moved position, and technological concerns, including maintaining connectivity. Despite this, 88% of participants stated that these concerns would not stop them from using the device in the future.

**Table 5:** Benefits and concerns of home cardiotocography monitoring, cited by participants.

Acceptability	Number (%)
**Benefits**	
Provides reassurance	26 (52%)
Saves time/travel	17 (34%)
Enables convenience	16 (32%)
Saves money	15 (30%)
Feels comforting	14 (28%)
Easy to use	8 (16%)
Reduces hospital attendance	8 (16%)
Reduces anxiety	7 (14%)
Facilitates confidence	4 (8%)
Improves access to care	2 (4%)
Reduces childcare concerns	2 (4%)
Aids with baby bonding	1 (2%)
Allows more time with family	1 (2%)
**Concerns**	
Locating the fetal heartbeat	16 (32%)
Technological concerns and connectivity	13 (26%)
No concerns	7 (14%)
Could exacerbate anxiety	7 (14%)
Time commitment	6 (12%)
Requires support	3 (6%)
Uncomfortable to monitor at home	2 (4%)
Device straps inadequate	2 (4%)
Requires a high degree of digital literacy	2 (4%)
Requires too much concentration	1 (2%)
Reduces in-person appointments	1 2%)
Concern over accuracy to conventional CTG	1 (2%)

## Discussion

### Main findings

This prospective study was designed to comprehensively evaluate the feasibility, acceptability, usability and safety of home CTG, whilst also identifying factors which influence technical adequacy. A total of 204 remote monitoring sessions were completed, with 82.4% (95% CI, 76.5%-87.0%) of sessions achieving 20 minutes of continuous, interpretable CTG monitoring. The mean length of monitoring was far greater than the benchmark designed for achieving competency at 34.3 ± 16.8 minutes. However, the number of sessions was not statistically significant for improving competency or duration of monitoring (p = 0.19 and p = 0.16 respectively). Participant adherence remained high throughout the study, within 8.2 ± 15.7 minutes from the pre-arranged start time. There was a statistically significant reduction in pre- and post-study anxiety (37.5 ± 11.7 versus 31.6 ± 12.0, p = 0.0004), including directly prior to and following the first (35.7 ± 11.0 versus 30.4 ± 11.4, p = 0.01) and fifth monitoring sessions (32.8 ± 11.8 versus 27.3 ± 7.7, p = 0.007). In the logistic analysis, no maternal characteristics were associated with improved monitoring competency. Sensitivity analysis demonstrated a high degree of numerical consistency across different modelling approaches. Thirteen remote sessions from 10 participants required referral to maternity triage for assessment and no pre-specified maternal-perinatal adverse safety outcomes occurred relating to device usage. Very few device-related technical errors occurred, with a single prolonged transmission delay, but no transmission failures. Acceptability and usability were rated highly among participants. Commonly cited benefits of home CTG included: reassurance, time saving and convenience, whilst concerns arose over difficulty in locating the fetal heartbeat and connectivity. Overall, 78% of participants were happy to only have home CTGs in a future pregnancy, rather than traditional outpatient CTGs.

### Strengths and limitations

Strengths of this study include the high number of home monitoring sessions, the repeated-measure design, the inclusion of a heterogeneous high-risk population and the detailed psychological assessment. A total of 204 remote monitoring sessions represents a substantial increase compared with our previous evaluation (59 sessions), thereby improving the robustness of the feasibility outcomes [18]. Enabling participants to complete up to six monitoring sessions facilitated assessment of usability over time, as well as the influence of increasing patient understanding on data acquisition. Furthermore, evaluating home monitoring on a broad spectrum of high-risk conditions enhances the clinical relevance of this technology and supports its potential applicability across all maternity units. Additionally, the within-session and longitudinal assessment of anxiety provides a deeper granular insight into the potential psychological benefits of home CTG monitoring. This fosters a more patient-centred approach and suggests that home CTG may off both patient and clinical advantages. Finally, prior literature on home CTG has yet to perform detailed logistic regression analysis on their study cohorts. Our results provides a nuanced evaluation, enabling researchers to begin to understand the influence of participant characteristics on monitoring competency.

Limitations of the study include the modest sample size, lack of comparator group, variability in CTG completion and usage of self-reported measures. Firstly, the sample size of 50, whilst suitably within the remit of feasibility assessment, may limit statistical power and increase the risk type 2 error and overinterpretation of observed trends. Secondly, the absence of a concurrent comparator group limits the ability to assess the relative effectiveness of home CTG compared with standard in-clinic monitoring, particularly in relation to outcomes such as anxiety and safety. Future evaluation in a randomised controlled trial comparing home CTG with standard care, in a larger cohort, would be important to address this limitation. Thirdly, it is also important to note that participants willing to enrol in the study may have been more digitally motivated and willing to learn, potentiating a degree of selection bias and leading to overestimating device feasibility. Additionally, several participants did not complete the maximum sessions, either due to early delivery or study exit, potentially introducing bias in the longitudinal findings. To combat this, sensitivity analyses were performed using a standard logistic regression model that did not account for within-participant clustering. Results were highly consistent with those obtained from the primary mixed-effects logistic regression model, suggesting that the study findings were robust to alternative assumptions regarding the independence of observations. Additionally, the evaluation of anxiety and usability relied on self-reported measures which are additionally subject to reporting bias. Observed reductions in anxiety may therefore reflects increased familiarity with study procedures rather than a direct improvement in clinical status.

In addition, it should be noted that the sample size for this study was calculated to detect a difference in the anxiety outcome, rather than in the primary outcome. This was decided as no comparable prior data existed to support an a-priori power calculation for the primary outcome. However, it should be noted that this discrepancy may affect the precision of the primary outcome estimate and the statistical power available for the multivariable analysis examining the predictors of monitoring competency. We have therefore reported the 95% CI around the primary outcome estimate, and findings in the regression model are presented as exploratory, rather than as definitive evidence.

Finally mixed-effects logistic regression analysis was conducted with an events per parameter (EPP) ratio of approximately 7.2, which is below the traditionally recommended threshold of ≥10. This may increase the risk of overfitting and unstable coefficient estimates. However, this threshold has been increasingly debated, and methodological literature suggests that models with lower EPP (5–10) can still provide valid and informative results, particularly in exploratory or feasibility studies [31,32]. This is valid when the number of predictors is limited and clinically justified and the model is not used for prediction but for identifying potential associations. In this study, regression analyses were chosen a-priori and were hypothesis-driven. However, results were interpreted cautiously given the limited sample size and require confirmation in larger datasets with greater event counts to ensure model stability and generalisability. Reassuringly, the consistency of results across multiple logistic models provides additional certainty regarding the robustness of the current results.

The limited sample size also constrained the number of covariates that could be included in the regression model. Although pregnancy-related characteristics such as estimated fetal weight, fetal presentation, and amniotic fluid volume may plausibly influence CTG signal acquisition and may be valuable to assess in future studies, their inclusion in the current analysis would have further reduced the EPP ratio and increased the risk of overfitting. Furthermore, ultrasound assessments were performed as part of routine clinical care and were not scheduled to coincide with individual home monitoring sessions, limiting the accuracy of these variables as session-level predictors. Larger studies with greater statistical power and more contemporaneous ultrasound data may be better positioned to evaluate the influence of fetal and pregnancy-related characteristics on monitoring success and device performance. A larger dataset would also permit more detailed parameterisation of covariates within the regression model. For example, ethnicity was dichotomised in the current analysis to maintain an acceptable EPP ratio. While methodologically necessary, such categorisation may oversimplify the complex social, cultural, and structural factors that influence engagement with digital healthcare interventions and can limit the interpretability of findings. A larger sample size would enable the use of more granular ethnicity categories, facilitating a more nuanced exploration of potential differences in home monitoring engagement across population groups.

### Generalisability and target population boundaries

The eligibility criteria for this study was constructed to align with the regulatory and technical constraints of the home CTG device. Therefore, the study cohort represent a subgroup within a wider population of high-risk pregnancies. As such, the results should be interpreted within these boundaries. Firstly, women with a BMI > 35 kg/m^2^ were excluded due to regulatory thresholds set by the device manufactures, as maternal adipose tissue can alter the signal quality of the fetal heartrate transducer. Nonetheless, this group of women represent a substantial proportion of high-risk pregnancies, who may benefit from home CTG monitoring. Therefore, evaluation of monitoring technologies, validated for higher BMI’s, should be an important research priority. Non-English speaking women were excluded due to constraints of study procedures, relating to availability of translated materials and interpreter services. This reflects a common resource restriction with feasibility research, rather than an unsuitability of the home CTG device. Future implementation research should incorporate appropriately translated materials and care pathways supported by interpreters, ensuring to avoid widening any preexisting health inequalities in antenatal care. Women with multiple pregnancies and fetal anomalies were excluded, due to inherently greater difficulties with routine CTG monitoring and currently, home CTG devices have not been validated within these contexts. Similarly, women with Doppler abnormalities were excluded, as such findings typically require intensive hospital fetal surveillance and are at higher risk of CTG monitoring abnormalities, needing emergency intervention. It is recognised that several of the excluded groups, such as women with higher BMI’s and non-English speaking women represent cohorts attending the outpatient departments regularly and thus likely to have a clear clinical need for remote CTG surveillance. Extrapolating the home CTG evidence base to these populations, thought device adaption and the availability of translation and interpreter support represent a necessary and vital direction for future research, particularly upon considering wider implementation of home monitoring.

### Interpretation

Recent years have witnessed a notable increase in research interest surrounding the exploration and evaluation of devices that allow for ‘hospital at home care’, including home CTG. This trend is likely driven by escalating pressures on outpatient obstetric services, attributable in part to the growing prevalence of high-risk pregnancies. Within the context of rapid technological advancement, home CTG presents a potential solution to the logistical constraints associated with conventional in-clinic fetal monitoring. A number of studies have evaluated the feasibility of home fetal monitoring across diverse cohorts, including both for low-risk [33] and high-risk populations [34–36], with high degrees of success. A recent systematic review conducted by this research group, encompassing 39 studies on home CTG monitoring, identified 23 studies that primarily evaluated feasibility outcomes [17]. Across these studies, the proportion of monitoring episodes yielding interpretable remote data ranged from 74-100%, which correlates closely the reported primary outcome in this present study [17]. In addition, three studies included within this review evaluated device usability using the SUS, reporting scores between 76.5-88.1 out of 100 [37–39]. The findings presented here are concordant with these prior results, with a mean SUS score of 77.6.

Despite these promising findings, the existing feasibility literature demonstrates a relative paucity of data pertaining to technical performance and safety outcomes; both of which are critical considerations in determining the broader applicability of home CTG [17]. The present study sought to address this gap by descriptively characterising these parameters associated with home CTG use, within this particular cohort. Our results indicate that 6.4% of monitoring episodes necessitated referral to maternity triage for further assessment, a figure descriptively similar to the median incidence of abnormal CTG recordings (6.5%) reported in our prior systematic review [17].However, whilst descriptively similar, results are not directly comparable due to differences in study populations, monitoring protocols and referral thresholds. It is important to note that the safety protocol employed in this present study was deliberately conservative. Consequently, 54% (7/13) referred cases to triage were triggered by low STV values (<5ms), typically with the association with minor computerised CTG abnormalities (no episodes of high variation). Under current UK obstetric practice, such findings during in-clinic monitoring would typically prompt repeat CTG assessment after four hours, or the following day, rather than immediate referral. Had a similar protocol been applied within this cohort, specifically repeating CTG monitoring in the home setting, the rate of hospital referrals may have been reduced to approximately 2.9%. This lower rate may be more acceptable when considering the scalability of home CTG implementation. If this technology is considered for potential future integration into routine care, with the aim of replacing or supplementation hospital CTG monitoring, it is essential to acknowledge and mitigate the downstream impact of increased home-to-hospital referrals on healthcare services, particularly within maternity triage units. Aligning home monitoring protocols more closely with established in-clinic practices may represent a pragmatic approach to addressing this challenge. These considerations should also extend to monitoring sessions that fail to achieve a sustained continuous CTG recording. In the present study, poor monitoring competency was not considered an indication for hospital attendance; however, approximately 17% of participants experienced difficulties obtaining an adequate recording. If future implementation pathways require women with unsuccessful monitoring attempts to attend hospital for assessment, this could place a substantial additional burden on maternity services. Consequently, strategies to improve monitoring competency through targeted education, user support, and enhanced device usability should be prioritised. Furthermore, clear clinical pathways will be required to manage unsuccessful recordings while minimising unnecessary hospital attendances and maintaining patient safety.

The most recent and methodologically influential trial evaluating home CTG, the HoTeL trial, conducted by Bekker et al. (2023), examined 200 high-risk obstetric participants who were evenly randomised to either home monitoring or inpatient care [13]. The findings demonstrated non-inferiority of home CTG with respect to the composite adverse primary outcome. Moreover, synthesis of all trials on home CTG (n = 7), in our prior systematic review, albeit many far older, similarly indicated non-inferiority across a range of maternal, fetal and healthcare utilisation outcomes, however, minimal outcome standardisation was noted [17,40].

Despite demonstrating non-inferiority, it is important to note that, in many studies, the comparator group was inpatient admission. This differs from standard practice in the UK, where management of high-risk pregnancies more commonly involves structured outpatient surveillance rather than routine hospitalisation. Consequently, there remains a need for evidence evaluating how home CTG can be operationalised within contemporary NHS maternity pathways, prior to drawing definitive conclusions regarding its future integration. Importantly, the principal contribution of this present study was to begin to evaluate the feasibility and acceptability of implementing a home CTG monitoring pathway within a UK NHS setting across a range of high-risk conditions, rather than developing comparative clinical-effectiveness evidence. Across 204 home monitoring episodes, we were able to characterise key implementation outcomes including participant recruitment and retention, adherence to the monitoring protocol, successful completion and transmission of CTG recordings, factors influencing technical adequacy, rates of technical failure, and patient acceptability. These data address an important evidence gap not fully explored in previous feasibility and effectiveness studies and provide practical information regarding service delivery, resource requirements and pathway design within a contemporary NHS outpatient model. Such implementation evidence is essential before larger comparative effectiveness trials can be designed and undertaken within UK maternity services. Presently, this study represents the largest feasibility study on home CTG within the UK setting.

Furthermore, whilst demonstration of non-inferiority is a necessary and important benchmark, it may be insufficient in isolation to justify widespread implementation of home CTG. In this present study, usage of the device was associated with a statistically significant reduction in maternal anxiety, suggesting potential added value beyond purely clinical equivalence. However, as a single-arm study without a control group, we are unable to determine whether this reduction reflects a direct psychological effect of the monitoring device itself or rather may be influenced by the frequent clinical interaction embedded within the study pathway, such as the post-monitoring telephone calls to communicate monitoring results, or the natural progression of pregnancy over the study period, or perhaps a combination of several factors. Future research should seek to evaluate this outcome within a comparative trial framework against standard care, ideally incorporating a comparator arm matched for frequency of clinical contact, while also exploring the broader economic and environmental implications of home CTG monitoring. However, before a definitive UK trial is undertaken, further work is required to better understand the acceptability of home CTG among both pregnant women and healthcare professionals. Qualitative research may provide important insights into perceived benefits, barriers to adoption, trust in remote monitoring, and the practical challenges associated with integrating home CTG into routine maternity care. In addition, further pathway modelling is required to determine how home CTG could be incorporated into existing surveillance strategies for different high-risk pregnancy indications, including the identification of patient groups most likely to benefit and the development of appropriate escalation and governance frameworks.

Notably, all home CTG episodes in this study were monitored synchronously by a single member of the research team, who was also responsible for coordinating any urgent attendance to triage. Although feasible within a research setting, this model is resource-intensive and unlikely to be scalable in routine NHS maternity care without substantial reconfiguration of existing workforce models. Wider implementation would require addressing several operational questions. This includes but is not limited to: 1) determining clinical responsibility for remote-trace interpretation (e.g., digital midwives, obstetricians); 2) the need for clear governance, escalation and medicolegal frameworks for managing safety and defining accountability for missed, delayed or misinterpreted CTG traces; 3) staffing and workforce training, alongside the associated costings; and 4) determining the interoperability of remote technologies with existing electronic health records [41,42]. Consequently, a comprehensive programme of research encompassing clinical effectiveness, implementation science, health economics, clinical governance, and patient-centred outcomes is additionally required to establish the evidence base necessary for safe and sustainable adoption of home CTG in contemporary NHS maternity care.

In this study, assessment of maternal anxiety demonstrated a statistically significant reduction following overall device use, as well as immediately after the first and fifth monitoring sessions. In contrast, the reduction in anxiety following the third monitoring session did not reach statistical significance. One possible explanation for this pattern is that initial exposure to home CTG generates a pronounced reassurance effect, as participants become familiar with the device, perceive it as safe, and feel comfort from hearing the fetal heartbeat. By the third session, this effect may attenuate as the initial novelty diminishes, with participants entering a phase of ‘neutral adaptation’ in which anxiety levels stabilise. The subsequent re-emergence of a statistically significant reduction in anxiety by the fifth session and at study completion may reflect the development of greater user confidence, an enhanced sense of control, and reinforced reassurance through repeated use. These findings suggest a potentially non-linear trajectory in anxiety response to home CTG monitoring, which is well supported by established psychological theory [43–45]. However, further investigation in larger cohorts is required to more precisely characterise the psychological impact of home CTG monitoring and to validate these preliminary observations.

Regarding the logistic regression analysis, home monitoring competency was not primarily associated by traditional socio-demographic markers of digital inequality, such as education or ethnicity, nor was it associated with experiential-related factors, including maternal age and parity. This may, in part, be attributable to the standardised training program provided to all participants before device usage. This may have mitigated potential disparities in digital literacy and facilitated equitable acquisition of technical skills required for home monitoring. Furthermore, the consistency of findings across clustered and non-clustered sensitivity analyses suggests that within-participant correlation had minimal influence on estimated associations, supporting the robustness of the observed results. Although not statistically significant, increasing maternal age demonstrated a trend towards lower monitoring competency. This finding should be interpreted cautiously given the exploratory nature of the analysis. Nevertheless, it is plausible that age-related differences in confidence or engagement with digital technologies may have contributed to the observed direction of effect. Larger studies are required to determine whether this apparent association represents a true underlying relationship.

Whilst the influence of BMI on competency demonstrated non-significance, it should be noted that the true impact cannot be evaluated as participants with a BMI > 35 kg/m² were excluded due to device regulatory limitations. Consequently, these findings may underestimate the impact of higher BMI on device performance and technical competency. Overall, these findings do suggest that, within a structured training framework, home CTG monitoring may be broadly accessible across demographic groups, supporting its potential as an inclusive digital health intervention.

## Conclusion

This single-arm feasibility study demonstrates that home CTG monitoring for high-risk pregnancy monitoring is a feasible, acceptable and usable endeavour. Furthermore, no safety concerns were identified within this cohort. In an era of technological advancement, home monitoring has scope to reshape the delivery of obstetric antenatal care. Further research should seek to evaluate home CTG monitoring within a comparative trial framework against standard care, while also exploring the broader economic and environmental implications of home CTG monitoring. A multifaceted assessment may help to establish a more comprehensive evidence base for this form of monitoring, potentially supporting its wider adoption in contemporary maternity care.

## Supporting information

S1 Table Definitions for eligible high risk conditions. (DOCX)

S2 Table Study dataset. (XLSX)

S3 Table Multivariable logistic regression analysis demonstrating the association between maternal age and technical competency, when stratified by parity. (DOCX)

S4 Table Pre- and post-study questionnaire responses. (DOCX)
