# Supplementary material for: Home cardiotocography for high-risk obstetric care: A prospective observational feasibility study
Source: PLOS Digit Health. 2026 Nov 6;5(11):e0001823. doi: 10.1371/journal.pdig.0001823 (PMC13742941; doi:10.1371/journal.pdig.0001823)
Supplement: Supplementary file 1 [file pdig.0001823.s001.docx]

**S1 Table.** Definitions for eligible high risk conditions.

| Condition | | Definition |
| --- | --- | --- |
| Hypertensive disease | Gestational hypertension | New-onset high blood pressure (≥140/90mmHg) detected after 20 weeks of pregnancy without protein in the urine or other signs of pre-eclampsia |
|  | Pre-eclampsia | New-onset high blood pressure (≥140/90mmHg) detected after 20 weeks of pregnancy with significant protein in the urine with or without other signs of pre-eclampsia |
| Small for gestational age | | Estimated fetal weight below the 10^th^ percentile for their gestational age |
| Fetal growth restriction | | Estimated fetal weight or abdominal circumference below the 3^rd^ percentile for their gestational age |
| Recurrent reduced fetal movements | | Two or more episodes of reduced fetal movements requiring attendance to maternity triage during the index pregnancy |
| Preterm prelabour rupture of membranes | | Rupture of membranes prior to 37 weeks gestation |
| Previous history of stillbirth | | Previous history of stillbirth after 24 weeks gestation. |
| Obstetric cholestasis | | Pregnancy related itchiness, without a rash, accompanied by abnormal liver function tests and/or raised serum bile acids. |
