# Supplementary material for: Home cardiotocography for high-risk obstetric care: A prospective observational feasibility study
Source: PLOS Digit Health. 2026 Nov 6;5(11):e0001823. doi: 10.1371/journal.pdig.0001823 (PMC13742941; doi:10.1371/journal.pdig.0001823)
Supplement: Supplementary file 2 [file pdig.0001823.s003.docx]

**S3 Table.** Multivariable logistic regression analysis demonstrating the association between maternal age and technical competency, when stratified by parity.

| **Variable** | **Primary GLMM OR (95% CI)** | **P value** | **GLM (no clustering) OR (95% CI)** | **P value** | **GLMM (BOBYQA) OR (95% CI)** | **P value** |
| --- | --- | --- | --- | --- | --- | --- |
| Age | 0.91 (0.82-1.01) | 0.09 | 0.92 (0.85-1.00) | 0.06 | 0.91 (0.81-1.01) | 0.09 |
| BMI | 1.00 (0.88- 1.14) | 1.00 | 1.01 (0.90- 1.12) | 0.93 | 1.00 (0.87- 1.15) | 1.00 |
| Ethnicity | 1.14 (0.41-3.16) | 0.80 | 1.15 (0.50-2.68) | 0.74 | 1.14 (0.38-3.31) | 0.80 |
| Parity | 1.45 (0.53-3.97) | 0.47 | 1.45 (0.64-3.26) | 0.37 | 1.45 (0.50-4.31) | 0.47 |
| Education | 1.49 (0.46-4.85) | 0.51 | 1.37 (0.53-3.55) | 0.51 | 1.49 (0.44-5.41) | 0.60 |

BMI, body mass index; BOBYQA, Bound Optimisation BY Quadratic Approximation; CI, confidence interval; GLM, generalised linear model; GLMM, generalised linear mixed model; OR, odds ratio.
