# Supplementary material for: Home cardiotocography for high-risk obstetric care: A prospective observational feasibility study
Source: PLOS Digit Health. 2026 Nov 6;5(11):e0001823. doi: 10.1371/journal.pdig.0001823 (PMC13742941; doi:10.1371/journal.pdig.0001823)
Supplement: Supplementary file 3 [file pdig.0001823.s004.docx]

**S4 Table**. Pre- and post-study questionnaire responses.

|  | **Pre-study** | **Post-study** | **P Value** |
| --- | --- | --- | --- |
| **Affective attitude** | | | |
| I would like to use/ liked using this device at home | Strongly agree: 25 (50%)  Agree: 24 (48%)  Neutral: 1 (2%)  Disagree: 0 (0%)  Strongly disagree: 0 (0%) | Strongly agree: 21 (42%)  Agree: 17 (34%)  Neutral: 8 (16%)  Disagree: 4 (8%)  Strongly disagree: 0 (0%) | **0.002** |
| **Burden** | | | |
| How much effort do you /did you think it takes to use the device at home | No effort at all: 5 (10%)  A little effort: 17 (34%)  Neutral: 18 (36%)  A lot of effort: 7 (14%)  Huge effort: 3 (6%) | No effort at all: 12 (24%)  A little effort: 24 (48%)  Neutral: 6 (12%)  A lot of effort: 6 (12%)  Huge effort: 2 (4%) | **0.002** |
| **Ethicality** | | | |
| Home monitoring during pregnancy fits well with my values | Strongly agree: 17 (34%)  Agree: 26 (52%)  Neutral: 7 (14%)  Disagree: 0 (0%)  Strongly disagree: 0 (0%) | Strongly agree: 21 (42%)  Agree: 19 (38%)  Neutral: 10 (20%)  Disagree: 0 (0%)  Strongly disagree: 0 (0%) | 0.42 |
| **Perceived effectiveness** | | | |
| Home monitoring would/did improve my access to pregnancy care | Strongly agree: 15 (30%)  Agree: 23 (46%)  Neutral: 12 (24%)  Disagree: 0 (0%)  Strongly disagree: 0 (0%) | Strongly agree: 18 (36%)  Agree: 21 (42%)  Neutral: 6 (12%)  Disagree: 5 (10%)  Strongly disagree: 0 (0%) | 0.81 |
| Home monitoring would/did make me feel safe in my pregnancy | Strongly agree: 17 (34%)  Agree: 27 (54%)  Neutral: 6 (12%)  Disagree: 0 (0%)  Strongly disagree: 0 (0%) | Strongly agree: 21 (42%)  Agree: 21 (42%)  Neutral: 6 (12%)  Disagree: 1 (2%)  Strongly disagree: 1 (2%) | 0.56 |
| Home monitoring would/did reassure me about my baby’s wellbeing | Strongly agree: 26 (52%)  Agree: 19 (38%)  Neutral: 5 (10%)  Disagree: 0 (0%)  Strongly disagree: 0 (0%) | Strongly agree: 24 (48%)  Agree: 18 (36%)  Neutral: 4 (8%)  Disagree: 3 (6%)  Strongly disagree: 1 (2%) | 0.55 |
| Home monitoring alone would be more flexible than hospital monitoring | Strongly agree: 19 (38%)  Agree: 20 (40%)  Neutral: 10 (20%)  Disagree: 1 (2%)  Strongly disagree: 0 (0%) | Strongly agree: 15 (30%)  Agree: 17 (34%)  Neutral: 12 (24%)  Disagree: 5 (10%)  Strongly disagree: 1 (2%) | 0.12 |
| **Intervention coherence** | | |  |
| Home monitoring would be/is easier to do than attending the same monitoring in hospital | Strongly agree: 18 (36%)  Agree: 21 (42%)  Neutral: 11 (22%)  Disagree: 0 (0%)  Strongly disagree: 0 (0%) | Strongly agree: 20 (40%)  Agree: 12 (24%)  Neutral: 10 (20%)  Disagree: 7 (14%)  Strongly disagree: 1 (2%) | 0.12 |
| Home monitoring would/did improve my understanding of my baby’s wellbeing | Strongly agree: 16 (32%)  Agree: 28 (56%)  Neutral: 6 (12%)  Disagree: 0 (0%)  Strongly disagree: 0 (0%) | Strongly agree: 21 (42%)  Agree: 17 (34%)  Neutral: 8 (16%)  Disagree: 4 (8%)  Strongly disagree: 0 (0%) | 0.12 |
| **Self-efficacy** | | |  |
| I would/did have more control of my pregnancy care by monitoring at home | Strongly agree: 10 (20%)  Agree: 25 (50%)  Neutral: 14 (28%)  Disagree: 1 (2%)  Strongly disagree: 0 (0%) | Strongly agree: 17 (34%)  Agree: 16 (32%)  Neutral: 13 (26%)  Disagree: 4 (8%)  Strongly disagree:0 (0%) | 0.67 |
| **Opportunity costs** | | |  |
| Only having this home monitoring compared to the same hospital monitoring would likely save me money | Strongly agree: 17 (34%)  Agree: 17 (34%)  Neutral: 13 (26%)  Disagree: 3 (6%)  Strongly disagree: 0 (0%) | Strongly agree: 19 (38%)  Agree: 17 (34%)  Neutral: 10 (20%)  Disagree: 4 (8%)  Strongly disagree: 0 (0%) | 0.66 |
| Only having this home monitoring compared to the same hospital monitoring would allow me to spend more time with my loved ones | Strongly agree: 13 (26%)  Agree: 21 (42%)  Neutral: 12 (24%)  Disagree: 4 (8%)  Strongly disagree: 1 (2%) | Strongly agree: 17 (34%)  Agree: 23 (46%)  Neutral: 7 (14%)  Disagree: 1 (2%)  Strongly disagree: 2 (2%) | 0.17 |
| Only having home monitoring would likely reduce the amount of time I attend the hospital for the same monitoring. | Strongly agree: 18 (36%)  Agree: 24 (48%)  Neutral: 7 (14%)  Disagree: 1 (2%)  Strongly disagree: 0 (0%) | Strongly agree: 19 (38%)  Agree: 24 (48%)  Neutral: 5 (6%)  Disagree: 2 (4%)  Strongly disagree: 0 (0%) | 0.77 |
| **General acceptability** | | |  |
| Overall, how acceptable do you find the home monitoring device |  | Completely acceptable: 27 (54%)  Acceptable: 15 (30%)  Neutral: 7 (14%)  Unacceptable: 1 (2%)  Completely unacceptable: 0 (0%) |  |
| **Future considerations** | | |  |
| I would be happy to only use this device rather than coming to hospital for the same test during my next pregnancy | Strongly agree: 17 (34%)  Agree: 18 (36%)  Neutral: 13 (26%)  Disagree: 2 (4%)  Strongly disagree: 0 (0%) | Strongly agree: 22 (44%)  Agree: 17 (34%)  Neutral: 6 (12%)  Disagree: 5 (10%)  Strongly disagree: 0 (0%) | 0.36 |
