# Supplementary material for: Home cardiotocography for high-risk obstetric care: A prospective observational feasibility study
Source: PLOS Digit Health. 2026 Nov 6;5(11):e0001823. doi: 10.1371/journal.pdig.0001823 (PMC13742941; doi:10.1371/journal.pdig.0001823)
Supplement: Supplementary file 4 [file pdig.0001823.s006.docx]

Department of Applied Health Sciences

University of Birmingham

16^th^ September 2026

RE: Manuscript PDIG-D-26-01085

**Dear Dr Chenxi Yang**

Thank you very much for your invaluable input and for considering our article for publication in PLOS Digital Health. Please find the reviewer comments below, with our responses following.

**Reviewer 1**

This is a well-conducted, methodologically ambitious feasibility study evaluating home cardiotocography (CTG) in a UK high-risk obstetric population. The study addresses an important and timely topic, given the increasing pressures on antenatal outpatient services and the NHS’s strategic direction toward community-based and digitally enabled care.

Thank you kindly for your positive comment.

However, several methodological and interpretative issues require attention before the manuscript can be accepted for publication. These include concerns about the absence of a control group, sample size justification, external validity, adverse event ascertainment, regression modelling strategy, and over-optimism in the discussion regarding implementation readiness. The authors are encouraged to address the following comments, which are intended to strengthen the manuscript’s scientific rigour and clinical utility.

**Comment 1:** Lack of a control group limits interpretation; conclusions should be aligned with feasibility design. This is a single-arm feasibility study without a concurrent control arm (e.g., routine outpatient CTG). However, the Discussion section repeatedly invokes non-inferiority and safety-related inferences (e.g., lines 343–345, 777–779), which extend beyond the evidentiary scope of a feasibility investigation. I recommend that the authors clearly distinguish between feasibility and comparative effectiveness, restrict safety-related statements to descriptive findings, and avoid overextending them as comparative evidence.

Thank you for this important comment. We have revised the manuscript throughout to ensure safety-related statements are restricted to descriptive findings, and that feasibility and comparative effectiveness are clearly distinguished. Specific changes are listed below:

- **Abstract:** The statement that home CTG was "safe" now reads that monitoring was "feasible, acceptable, and usable, with no safety concerns identified in this single-arm cohort." This can be found on lines 53-54 of the manuscript with tracked changes.
- **Author Summary:** Has been revised to remove the term "safe endeavour" and rephrased in favour of purely descriptive language "without evidence of safety concerns". This can be found on line 66 of the manuscript with tracked changes.
- **Methods (reviewer's cited lines 343–345):** This passage previously referenced "comparative safety" and being "powered" for safety comparisons, even while disclaiming them. We have removed this vocabulary entirely. The text now states that descriptively collected safety data was used to identify potential safety concerns and inform the design of future studies. This can be found on lines 347-350 of the manuscript with tracked changes.
- **Discussion (reviewer's cited lines 777–779):** We have rephrased to state plainly that the principal contribution of this study was to evaluate feasibility and acceptability of home CTG within a UK NHS setting, rather than developing comparative clinical-effectiveness evidence. This can be found on lines 825-828 of the manuscript with tracked changes.
- **Discussion:** On further review, we identified an additional passage in which our triage referral rate (6.4%) was described as "closely comparable" to a rate reported in our prior systematic review, framed as "contributing further evidence" supporting clinical integration. We have revised this to make clear the comparison is descriptive only, noting explicitly that the two studies are not directly comparable given differences in population, protocol, and referral thresholds. This can be found on lines 779-781 of the manuscript with tracked changes.
- **Conclusion:** Has been revised to explicitly identify the study as a "single-arm feasibility study" and that ‘no safety concerns were identified within this cohort’, rather than saying home CTG monitoring is ‘safe’. This can be found on lines 931-934 of the manuscript with tracked changes.

**Comment 2:** Sample size calculation does not align with the primary outcome, affecting statistical robustness of multivariable analysis. The sample size was powered to detect a difference in anxiety scores (lines 401–403), whereas the primary outcome was defined as the proportion of sessions achieving ≥20 minutes of interpretable CTG. This discrepancy may compromise the statistical power for the primary endpoint. Moreover, the events-per-parameter (EPP) ratio in the multivariable logistic regression model was approximately 7.2, below the traditional recommended threshold (≥10). The authors should acknowledge this limitation transparently, consider reporting the precision of the primary outcome estimate (e.g., confidence interval width), and avoid drawing definitive "no association" conclusions from non-significant findings in the regression model.

Thank you for this important comment. We confirm that the sample size calculation (42 participants, 90% power) was based on detecting a difference in the secondary anxiety outcome, as no comparable prior data existed to support a formal a-priori power calculation for the primary outcome (achievement of 20 minutes of continuous, interpretable CTG monitoring). We have addressed the reviewer's three specific recommendations as follows:

- **Transparent acknowledgement of the limitation:** We have added an explicit statement to the Strengths and Limitations section naming this discrepancy directly, explaining that the study was powered around the secondary anxiety outcome rather than the primary outcome, and that no prior data were available to support powering the primary outcome itself. This can be found on lines 680-687 of the manuscript with the tracked changes.
- **Reporting the precision of the primary outcome estimate:** We have added the 95% confidence interval around the primary outcome estimate in both the Abstract and Results (82.4%, 95% CI 76.5%–87.0%), so readers can directly assess the precision of this estimate. This can be found on lines 38, 449 and 613 of the manuscript with the tracked changes.
- **Avoiding definitive "no association" conclusions:** We have replaced language describing maternal characteristics as simply "not associated" with monitoring competency, in the Abstract and Discussion sections, with language specifying "no *statistically significant* association" and explicitly framing this as an exploratory analysis. This can be found on line 43 and 687 of the manuscript with tracked changes. The ‘no statistically significant association’ and exploratory nature of the multivariable analysis was already pre-written on lines 322 and 694 of the manuscript with tracked changes.
- Information regarding the EPP ration of approximately 7.2 already exists in the Limitations section of the Discussion. Information regarding its implications for estimate stability, and the corresponding need for confirmation in larger, adequately powered datasets was originally written. Regarding the reviewers comment, we felt an additional sentence to further address this would be repetitive of this existing discussion. Information can be found on lines 689-701.

**Comment 3:** Exclusion criteria significantly limit generalisability; discussion of target populations requires expansion. The study excluded women with BMI >35 kg/m², non-English speakers, multiple pregnancies, and fetal anomalies—groups that often represent the highest clinical need and greatest potential benefit from remote monitoring. Although the authors briefly note the BMI limitation (lines 857–860), the broader implications of other exclusion criteria for external validity are insufficiently discussed. I recommend adding a dedicated section on "Generalisability and target population boundaries" to clarify the scope of populations to which the current evidence can be applied.

Thank you for this important comment. We agree that the implications of our exclusion criteria for external validity warrant a larger discussion beyond the brief note on BMI in the current Limitations section. As suggested, we have added a new, dedicated Discussion subsection, "Generalisability and target population boundaries," placed immediately after the Strengths and Limitations section and before the Interpretation section. This can be found on lines 723-750 of the manuscript with tracked changes.

**Comment 4:** Adverse event ascertainment and technical failure classification lack systematic operational definitions. The authors report the frequency of triage attendances prompted by home CTG, but it is unclear whether all adverse events—including device-related skin reactions, psychological distress, or delayed care—were systematically captured. Furthermore, technical failures such as "device self-termination" and "signal loss" are not classified by attributable cause (hardware malfunction, user error, or environmental factors). The authors should specify the standardised adverse event recording process and provide a more granular classification of technical failures (e.g., error codes, user logs, root cause analysis) to enhance reproducibility and inform device refinement.

We thank the reviewer for highlighting the need for greater transparency here. We have specified in the Methods how safety and technical issues were ascertained. This can be found on lines 336-351 of the revised manuscript with tracked changes. In the Results, we have added further classification information regarding the reported technical device issues. This can be found on lines 573-582 of the revised manuscript with tracked changes.

**Comment 5:** Discussion overstates implementation readiness while underplaying resource and governance challenges. Although the authors acknowledge that synchronous monitoring by a single researcher is unlikely to be scalable (lines 810–812), the overall Discussion remains overly optimistic toward adoption. Key implementation considerations are insufficiently addressed, including: (1) who would assume responsibility for real-time trace interpretation (e.g., digital midwives, obstetricians, or centralised monitoring hubs); (2) potential medicolegal liabilities and governance frameworks; (3) interoperability with existing electronic health records and maternity information systems; and (4) cost implications and workforce training requirements. A more balanced discussion of these operational realities is essential to enhance the manuscript's value for policymakers and service planners.

We thank the reviewer for this observation and agree the Discussion needed a more balanced treatment of implementation barriers. We have revised the relevant paragraph including to address the gaps raised in your comment. This can be found on lines 867-885 of the revised manuscript with tracked changes.

**Reviewer 2**

**Comment 1:** While the significant reduction in STAI scores is a positive finding, interpreting it is tricky without a control group receiving routine outpatient care. It isn't clear whether the lower anxiety stems from the monitoring device itself, natural progression of pregnancy, or the "high-touch" nature of the intervention (such as the midwife follow-up calls after each session). I suggest tempering the assertions in the Abstract and Discussion about the direct psychological effects of home CTG. Please include a note acknowledging that the frequent contact and clinical engagement during the study may have contributed to the lower anxiety levels.

We thank the reviewer for this insightful comment. We agree that, without a control group receiving routine outpatient care, we cannot truly confirm that the observed reduction in maternal anxiety is specifically related to the monitoring device itself. We have tempered this in the Discussion, as suggested. This can be found on lines 846-851 and of the manuscript with tracked changes.
