# Supplementary material for: Home cardiotocography for high-risk obstetric care: A prospective observational feasibility study
Source: PLOS Digit Health. 2026 Nov 6;5(11):e0001823. doi: 10.1371/journal.pdig.0001823 (PMC13742941; doi:10.1371/journal.pdig.0001823)
Supplement: Peer review history

# Peer review: Decision Letter 0

**DOI:** 10.1371/journal.pdig.0001823.r001

**Authors:** Hongxing Luo, Chenxi Yang

9 Sep 2026

Response to Reviewers Revised Manuscript with Track Changes Manuscript**Journal Requirements:** **Additional Editor Comments (if provided):** **Reviewers' Comments:**

**Comments to the Author**

1. Does this manuscript meet PLOS Digital Health’s publication criteria?

Reviewer #1: Yes

Reviewer #2: Yes

**********

2. Has the statistical analysis been performed appropriately and rigorously?

Reviewer #1: Yes

Reviewer #2: Yes

**********

3. Have the authors made all data underlying the findings in their manuscript fully available (please refer to the Data Availability Statement at the start of the manuscript PDF file)?

The PLOS Data policy

Reviewer #1: Yes

Reviewer #2: Yes

**********

4. Is the manuscript presented in an intelligible fashion and written in standard English?

Reviewer #1: Yes

Reviewer #2: Yes

**********

Reviewer #1: This is a well-conducted, methodologically ambitious feasibility study evaluating home cardiotocography (CTG) in a UK high-risk obstetric population. The study addresses an important and timely topic, given the increasing pressures on antenatal outpatient services and the NHS’s strategic direction toward community-based and digitally enabled care.

However, several methodological and interpretative issues require attention before the manuscript can be accepted for publication. These include concerns about the absence of a control group, sample size justification, external validity, adverse event ascertainment, regression modelling strategy, and over-optimism in the discussion regarding implementation readiness. The authors are encouraged to address the following comments, which are intended to strengthen the manuscript’s scientific rigour and clinical utility.

Comment 1: Lack of a control group limits interpretation; conclusions should be aligned with feasibility design

This is a single-arm feasibility study without a concurrent control arm (e.g., routine outpatient CTG). However, the Discussion section repeatedly invokes non-inferiority and safety-related inferences (e.g., lines 343–345, 777–779), which extend beyond the evidentiary scope of a feasibility investigation. I recommend that the authors clearly distinguish between feasibility and comparative effectiveness, restrict safety-related statements to descriptive findings, and avoid overextending them as comparative evidence.

Comment 2: Sample size calculation does not align with the primary outcome, affecting statistical robustness of multivariable analysis

The sample size was powered to detect a difference in anxiety scores (lines 401–403), whereas the primary outcome was defined as the proportion of sessions achieving ≥20 minutes of interpretable CTG. This discrepancy may compromise the statistical power for the primary endpoint. Moreover, the events-per-parameter (EPP) ratio in the multivariable logistic regression model was approximately 7.2, below the traditional recommended threshold (≥10). The authors should acknowledge this limitation transparently, consider reporting the precision of the primary outcome estimate (e.g., confidence interval width), and avoid drawing definitive "no association" conclusions from non-significant findings in the regression model.

Comment 3: Exclusion criteria significantly limit generalisability; discussion of target populations requires expansion

The study excluded women with BMI >35 kg/m², non-English speakers, multiple pregnancies, and fetal anomalies—groups that often represent the highest clinical need and greatest potential benefit from remote monitoring. Although the authors briefly note the BMI limitation (lines 857–860), the broader implications of other exclusion criteria for external validity are insufficiently discussed. I recommend adding a dedicated section on "Generalisability and target population boundaries" to clarify the scope of populations to which the current evidence can be applied.

Comment 4: Adverse event ascertainment and technical failure classification lack systematic operational definitions

The authors report the frequency of triage attendances prompted by home CTG, but it is unclear whether all adverse events—including device-related skin reactions, psychological distress, or delayed care—were systematically captured. Furthermore, technical failures such as "device self-termination" and "signal loss" are not classified by attributable cause (hardware malfunction, user error, or environmental factors). The authors should specify the standardised adverse event recording process and provide a more granular classification of technical failures (e.g., error codes, user logs, root cause analysis) to enhance reproducibility and inform device refinement.

Comment 5: Discussion overstates implementation readiness while underplaying resource and governance challenges

Although the authors acknowledge that synchronous monitoring by a single researcher is unlikely to be scalable (lines 810–812), the overall Discussion remains overly optimistic toward adoption. Key implementation considerations are insufficiently addressed, including: (1) who would assume responsibility for real-time trace interpretation (e.g., digital midwives, obstetricians, or centralised monitoring hubs); (2) potential medicolegal liabilities and governance frameworks; (3) interoperability with existing electronic health records and maternity information systems; and (4) cost implications and workforce training requirements. A more balanced discussion of these operational realities is essential to enhance the manuscript's value for policymakers and service planners.

Reviewer #2: - While the significant reduction in STAI scores is a positive finding, interpreting it is tricky without a control group receiving routine outpatient care. It isn't clear whether the lower anxiety stems from the monitoring device itself, natural progression of pregnancy, or the "high-touch" nature of the intervention (such as the midwife follow-up calls after each session). I suggest tempering the assertions in the Abstract and Discussion about the direct psychological effects of home CTG. Please include a note acknowledging that the frequent contact and clinical engagement during the study may have contributed to the lower anxiety levels.

**********

what does this mean?). If published, this will include your full peer review and any attached files.

**Do you want your identity to be public for this peer review?**  If you choose “no”, your identity will remain anonymous but your review may still be made public.

For information about this choice, including consent withdrawal, please see our Privacy Policy

Reviewer #1: No

Reviewer #2: No

**********

**Figure resubmission:**

**Reproducibility:**

To enhance the reproducibility of your results, we recommend that authors of applicable studies deposit laboratory protocols in protocols.io, where a protocol can be assigned its own identifier (DOI) such that it can be cited independently in the future. Additionally, PLOS ONE offers an option to publish peer-reviewed clinical study protocols. Read more information on sharing protocols at https://plos.org/protocols?utm_medium=editorial-email&utm_source=authorletters&utm_campaign=protocols

# Author comment: Author response to Decision Letter 1

**DOI:** 10.1371/journal.pdig.0001823.r002

21 Sep 2026

Attachment Submitted filename: Response to reviewer comments.docx

# Peer review: Decision Letter 1

**DOI:** 10.1371/journal.pdig.0001823.r003

**Authors:** Hongxing Luo, Chenxi Yang

24 Oct 2026

Home cardiotocography for high-risk obstetric care: a prospective observational feasibility study

PDIG-D-26-01085R1

Dear Dr Le Vance,

We are pleased to inform you that your manuscript 'Home cardiotocography for high-risk obstetric care: a prospective observational feasibility study' has been provisionally accepted for publication in PLOS Digital Health.

Before your manuscript can be formally accepted you will need to complete some formatting changes, which you will receive in a follow-up email from a member of our team.

Please note that your manuscript will not be scheduled for publication until you have made the required changes, so a swift response is appreciated.

IMPORTANT: The editorial review process is now complete. PLOS will only permit corrections to spelling, formatting or significant scientific errors from this point onwards. Requests for major changes, or any which affect the scientific understanding of your work, will cause delays to the publication date of your manuscript.

If your institution or institutions have a press office, please notify them about your upcoming paper to help maximize its impact. If they'll be preparing press materials, please inform our press team as soon as possible -- no later than 48 hours after receiving the formal acceptance. Your manuscript will remain under strict press embargo until 2 pm Eastern Time on the date of publication. For more information, please contact digitalhealth@plos.org.

Thank you again for supporting Open Access publishing; we are looking forward to publishing your work in PLOS Digital Health.

Best regards,

Chenxi Yang, Ph.D.

Academic Editor

PLOS Digital Health

***********************************************************

**Additional Editor Comments (if provided):**

Dear authors, congratulations on the study! We look forward to more high-quality studies from your group.

**Reviewer Comments (if any, and for reference):**

Reviewer's Responses to Questions

**Comments to the Author**

Reviewer #1: All comments have been addressed

Reviewer #2: All comments have been addressed

Reviewer #3: All comments have been addressed

**********

publication criteria?

Reviewer #1: Yes

Reviewer #2: Yes

Reviewer #3: Yes

**********

3. Has the statistical analysis been performed appropriately and rigorously?

Reviewer #1: Yes

Reviewer #2: Yes

Reviewer #3: Yes

**********

4. Have the authors made all data underlying the findings in their manuscript fully available (please refer to the Data Availability Statement at the start of the manuscript PDF file)?

The PLOS Data policy

Reviewer #1: Yes

Reviewer #2: Yes

Reviewer #3: Yes

**********

5. Is the manuscript presented in an intelligible fashion and written in standard English?

Reviewer #1: Yes

Reviewer #2: Yes

Reviewer #3: Yes

**********

Reviewer #1: (No Response)

Reviewer #2: The author has addressed the comments raised.

Reviewer #3: I have reviewed the revised manuscript and the authors' point-by-point response. My previous concerns have been fully addressed. I recommend acceptance.

**********

what does this mean?). If published, this will include your full peer review and any attached files.

**Do you want your identity to be public for this peer review?**  If you choose “no”, your identity will remain anonymous but your review may still be made public.

For information about this choice, including consent withdrawal, please see our Privacy Policy

Reviewer #1: No

Reviewer #2: No

Reviewer #3: No

**********
